# Maximal point‐polyserial correlation for non‐normal random distributions

**DOI:** 10.1111/bmsp.12362

**Published:** 2024-10-22

**Authors:** Alessandro Barbiero

**Affiliations:** ^1^ Department of Economics, Management and Quantitative Methods Università degli Studi di Milano Milan Italy

**Keywords:** attainable correlations, biserial correlation, discretization, latent variable, non‐normal distribution

## Abstract

We consider the problem of determining the maximum value of the point‐polyserial correlation between a random variable with an assigned continuous distribution and an ordinal random variable with k categories, which are assigned the first k natural values 1,2,…,k, and arbitrary probabilities $p_{i}$. For different parametric distributions, we derive a closed‐form formula for the maximal point‐polyserial correlation as a function of the $p_{i}$ and of the distribution's parameters; we devise an algorithm for obtaining its maximum value numerically for any given k. These maximum values and the features of the corresponding k‐point discrete random variables are discussed with respect to the underlying continuous distribution. Furthermore, we prove that if we do not assign the values of the ordinal random variable a priori but instead include them in the optimization problem, this latter approach is equivalent to the optimal quantization problem. In some circumstances, it leads to a significant increase in the maximum value of the point‐polyserial correlation. An application to real data exemplifies the main findings. A comparison between the discretization leading to the maximum point‐polyserial correlation and those obtained from optimal quantization and moment matching is sketched.

## 1 INTRODUCTION

In behavioural, educational, and psychological studies, the observed variables are frequently measured using ordinal scales. For example, the Likert scale is widely used to measure responses in surveys, allowing respondents to express how much they agree or disagree with a particular statement or the level of satisfaction they show towards a product they bought or a service they experienced, in a (typically) five‐ or seven‐point scale (e.g., 1 = ‘completely disagree’ or ‘completely unsatisfied’, …, 5 = ‘completely agree’ or ‘completely satisfied’). These categorical ordinal variables can be treated as being discretized from an underlying continuous variable for degree of agreement on the statement or level of satisfaction (see, e.g., Bartholomew, 1980; Zhang et al., 2024). There are also many examples of quantitative variables that are discretized explicitly in social science studies, for instance, when asking questions about sensitive or personal quantitative attributes (e.g., income, alcohol consumption, time spent on social media), the non‐response rate may often be reduced by simply asking the respondent to select one of two very broad categories (e.g., under 50 K/over 50 K). When analysing these kinds of data, a common approach is to assign consecutive integer scores (CISs) to the ordered categories and proceed in the analysis as if the data had been measured on an interval scale with desired distributional properties (Norman, 2010); ‘Parametric statistics can be used with Likert data, with small sample sizes, with unequal variances, and with non‐normal distributions, with no fear of “coming to the wrong conclusion”.’ The most common choice for the distribution of the latent variables is the (multivariate) normal distribution because the dependence structure among them can be fully captured by the variance‐covariance matrix and each of its elements can be estimated using a bivariate normal distribution separately (see, e.g., chapter 6 in McNeil et al., 2015).

Let $X_{2}$ be an observed ordinal variable that depends on an underlying latent continuous random variable (RV) $Z_{2}$, and let $Z_{1}$ represent another observed continuous variable. It is typically assumed that the joint distribution of $Z_{1}$ and $Z_{2}$ is bivariate normal. The product moment correlation between $Z_{1}$ and $X_{2}$ is called the point‐polyserial correlation, while the correlation between $Z_{1}$ and $Z_{2}$ is called the polyserial correlation. As a particular case, if $X_{2}$ is a dichotomous random variable, we refer to them as point‐biserial and biserial correlations. The problem of estimating the polyserial correlation based on a bivariate sample was studied by Cox (1974), who derived the maximum likelihood estimator (MLE); in a multivariate setting, the problem was later addressed by Lee and Poon (1986), who used the classical Newton‐Raphson algorithm to produce the estimates and their standard errors; Olsson et al. (1982) derived the relationship between the polyserial and the point‐polyserial correlation and compared the MLE of polyserial correlation with a two‐step estimator and with a computationally convenient ad hoc estimator. Bedrick (1995) studied the attenuation of the correlation coefficient (the polyserial correlation) when one of the continuous variables is categorized. The attenuation is shown to depend critically on the distribution of the underlying latent variable and on the scores assigned to the categories. It is observed that the reduction in correlation can be substantially greater under exponential, double exponential, and t distributions than is expected assuming normality. However, attenuation becomes less severe as the number of categories increases, provided the category scores are carefully selected. In particular, equally spaced scores (e.g., 1,2,…,k) give reasonable protection against gross attenuation across a variety of distributions. On the problem of assigning scores to ordered categories, consult Ivanova and Berger (2001) and Fernández et al. (2020).

Demirtas and Hedeker (2016) and, later, Demirtas and Vardar‐Acar (2017) studied the relationship between the biserial and the point‐biserial correlations by devising an algorithm working for any underlying distribution other than the (bivariate) normal for the bivariate vector $\left(Z_{1},Z_{2}\right)$. The authors state that ‘it works for ordinal‐continuous data combinations, and so one can compute the polyserial correlation given the point‐polyserial correlation (or vice versa) when the relative proportions of the ordinal categories are specified’. The algorithm is based on the generation of a huge sample (of size, say, N=100,000) from a bivariate random vector $\left(Z_{1},Z_{2}\right)$ with assigned marginal distributions and dependence structure, implicitly induced by the method of Fleishman polynomials (Fleishman, 1978) for the construction of bivariate random vectors (Foldnes & Grønneberg, 2015). Although the numerical experiments carried out in Demirtas and Hedeker (2016) are said to produce negligible errors (when an analytical solution is also available), nevertheless the sampling error naturally induced by random simulation can hardly be controlled and contitutes an obstacle if one is interested in determining the range of the point‐polyserial correlation. Cheng and Liu (2016) derived the maximal point‐biserial correlation under several non‐normal distributions, namely, the uniform, Student's t, exponential, and a mixture of two normal distributions. They showed that the maximal point‐biserial correlation, depending on the non‐normal continuous distribution, may or may not be a function of the probability p that the dichotomous variable takes the value 1; it may be symmetric or non‐symmetric around p=.5. The relatively easy analytical derivation of (maximal) point‐biserial correlation relies on the (availability of expression for) moments of truncated continuous distributions.

It would be interesting to extend the results of this latter work to any k>2 while avoiding explicit or implicit assumptions about the dependence structure between the two continuous random variables and minimizing the impact of sampling errors, as seen in previous contributions. The procedures developed in Demirtas and Hedeker (2016), in fact, are able to compute the correlation between a continuous and a discretized RV (and the corresponding correlational change) when their distribution before discretization is fully specified and a dependence structure is implicitly or explicitly assumed.

The aim of this paper is to derive the expression for the maximal point‐polyserial correlation, i.e., the maximal linear correlation between a continuous random variable and an ordinal RV with k categories, for several continuous random distributions. Along with the normal distribution, several widely used non‐normal distributions are considered, namely uniform, exponential, Pareto, logistic, and power distributions. We will start with the general case (an ordinal random variable taking the values 1,2,…,k with corresponding probabilities $p_{i}$, i=1,2,…,k) and consider the particular case of equal‐probability support values ($p_{i}=1/k$ for all i=1,2,…,k), which is suitable for studying the limit behaviour of the maximal point‐polyserial correlation. We will calculate, among all the k‐point ordinal distributions, the one that maximizes the maximal point‐polyserial correlation with the assigned continuous RV. We will also investigate the situation where the k values of the discrete random variable are not predefined as 1,…,k but are instead assigned numerical scores aimed at maximizing the correlation itself.

The paper is structured as follows. Section 2 reviews some results on attainable correlations between two random variables with assigned margins. Section 3 synthesizes and integrates the main findings about the point‐biserial and point‐polyserial correlation under bivariate normality. Section 4, after formulating the optimization problem, investigates the main features of the optimal solution and the behaviour of the maximum point‐polyserial correlation under normal and several non‐normal distributions. Section 5 illustrates the main findings using a real data set. Section 6 hints at a possible application of the results on maximal point‐polyserial correlations in finding an optimal k‐point approximation of a continuous distribution. Section 7 concludes the paper with some final remarks.

## 2 ATTAINABLE CORRELATIONS

Before introducing useful results about attainable correlations, we must review the concepts of comonotonicity and countermonotonicity for a pair of RVs. Two RVs X and Y are said to be comonotonic if they admit as copula the Fréchet upper bound M(u,v)=min(u,v). Equivalently, two RVs are comonotonic if they are monotonically increasing functions of a single RV; in other words, X and Y are comonotonic if and only if (X,Y) is equal in distribution to (v(Z),w(Z)) for some RV Z and increasing functions v and w. These two equivalent definitions encompass any type of RV, including absolutely continuous and discrete ones. If a discrete RV and a continuous RV are comonotonic, we observe that when we move towards a larger category of the former, the latter takes on larger values with probability 1. Two RVs X and Y are said to be countermonotonic if they admit as copula the Fréchet lower bound W(u,v)=max(0,u+v−1). Equivalently, two RVs are countermonotonic if and only if (X,Y) is equal in distribution to (v(Z),w(Z)) for some RV Z and increasing function v and decreasing function w.

Although Pearson's correlation ρ between two random variables X and Y can theoretically take on any value between −1 and +1; however, when the marginal distributions of X and Y are assigned, it may generally not span the entire [−1,+1] interval and may not reach either its natural lower or upper bound. The constraint induced by assigning the marginal distributions typically reduces the range of Pearson's correlation to a narrower interval. In more detail (Fréchet, 1951; Hoeffding, 1940), the minimal and maximal attainable correlations that Pearson's ρ can reach form a closed interval $\left[\rho_{\min},\rho_{\max}\right]$, with $\rho_{\min}<0<\rho_{\max}$. The minimum correlation $\rho_{\min}$ is attained if and only if X and Y are countermonotonic; the maximum correlation $\rho_{\max}$ is attained if and only if X and Y are comonotonic. Moreover, $\rho_{\min}=-1$ if and only if X and −Y are of the same type, and $\rho_{\max}=1$ if and only if X and Y are of the same type. We recall that two RVs X and Y (or their random distributions) are said to be of the same type if there exist two constants a∈ℝ and $b\in {\mathbb{R}}^{+}$ such that $X\overset{d}{=}a+bY$; in other words, X and Y are RVs of the same type if they are a location‐scale transformation of each other. The bounds for ρ are computed as $\rho_{\min}=\text{cor}(F_{1}^{-1}(U),F_{2}^{-1}(1-U))$ and $\rho_{\max}=\text{cor}(F_{1}^{-1}(U),F_{2}^{-1}(U))$, where U is a standard uniform RV, $F_{1}$ and $F_{2}$ are the marginal distributions of RVs X and Y respectively, and $F_{1}^{-1}$ and $F_{2}^{-1}$ are their generalized inverses or quantile functions. It is often possible to determine analytically the minimum and maximum attainable correlations by using the two formulas above; otherwise, they can be computed numerically by resorting to the algorithm in Demirtas and Hedeker (2011). A correlation value ρ is said to be ‘feasible’ given the assigned margins $F_{1}$ and $F_{2}$ if it falls within $\left[\rho_{\min},\rho_{\max}\right]$.

**FIGURE 1:**
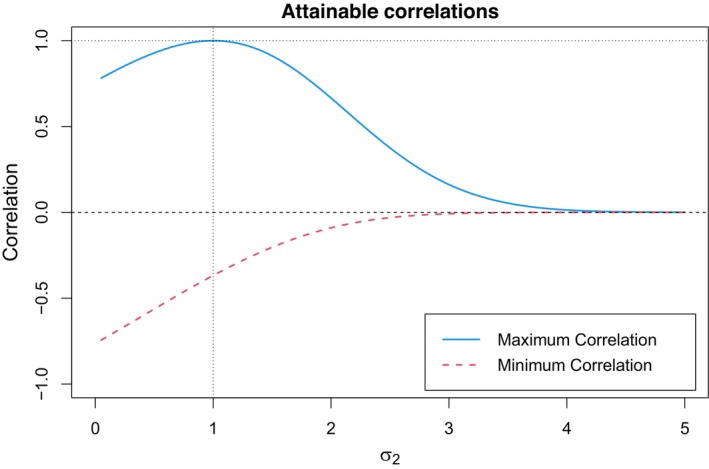
Attainable correlations between two lognormal RVs, $X∼ℒ\mathrm{34𝒩}(\mu_{1}=0,\sigma_{1}=1)$ and $Y∼ℒ\mathrm{34𝒩}(\mu_{2}=0,\sigma_{2})$.

This feature of Pearson's correlation, which is well known in the quantitative risk management field (Embrechts et al., 2002) but is often overlooked in other applied areas, represents a drawback and can lead to misinterpretations of its observed sample values. A typical example concerns two lognormal distributions with parameters $\mu_{1}=0$, $\sigma_{1}=1$ and $\mu_{2}=0$, $\sigma_{2}>0$. The two distributions are not of the same type unless $\sigma_{2}=\sigma_{1}$; the value of the minimal correlation is given by $\rho_{\min}=\frac{e^{-\sigma_{2}}-1}{\sqrt{\left(e-1)(e^{\sigma_{2}^{2}}-1\right)}}$, the value of the maximal correlation is $\rho_{\max}=\frac{e^{\sigma_{2}}-1}{\sqrt{\left(e-1)(e^{\sigma_{2}^{2}}-1\right)}}$. Therefore, if $\sigma_{2}=\sigma_{1}=1$, $\rho_{\max}=1$, and $\rho_{\min}\approx -0.368$; in fact, $X_{1}$ and $X_{2}$ are of the same type, but $X_{1}$ and $-X_{2}$ are not since the lognormal distribution is supported on ${\mathbb{R}}^{+}$ and is consequently asymmetric. For any $\sigma_{2}\neq \sigma_{1}$, $X_{1}$ and $X_{2}$ are not RVs of the same type, and the interval $\left[\rho_{\min},\rho_{\max}\right]$ tends to get narrower as $\sigma_{2}$ increases. For example, if $\sigma_{2}=2$, then we have that $\rho_{\max}=0.666$ and $\rho_{\min}\approx -0.090$; if $\sigma_{2}=4$, $\rho_{\max}\approx 0.014$, and $\rho_{\min}\approx 0.000$, then these latter values can lead the inadvertent researcher to claim that the two RVs are nearly uncorrelated, whereas the two RVs are indeed perfectly (positively/negatively) correlated! Figure 1 displays the maximum and minimum attainable correlations for the two lognormal RVs as functions of $\sigma_{2}$.

From the foregoing explanation, it is clear that if we consider a first RV with a continuous distribution and a second RV whose distribution is discrete, or is obtained by discretizing the former, then the maximum correlation cannot be +1, and the minimum correlation cannot be −1. This is because a discrete distribution can never be of the same type as a continuous distribution, simply due to the fact that the latter has a non‐countable support, whereas the former is defined over a finite or countable set.

The extreme values −1 and +1 can be potentially obtained only as limits when the cardinality of the support of the discrete RV increases and resembles a continuous one or when the continuous RV converges to a discrete RV when one of its parameters tends to a limiting value, as can occur in the case of a mixture of two normal distributions with the same variance (Cheng & Liu, 2016).

## 3 POINT‐POLYSERIAL CORRELATION UNDER NORMALITY

Let $\left(Z_{1},Z_{2}\right)$ be a bivariate standard normal RV, and let $X_{2}$ be a dichotomy of $Z_{2}$, with the point of dichotomy ω; thus, $X_{2}$ is a RV that takes a value of 1 when $Z_{2}\geq \omega$ and a value of 0 when $Z_{2}<\omega$. If φ(·) denotes the probability density function (PDF) of a standard normal RV and $P(X_{2}=1)=\int_{\omega }^{\infty }\varphi (y)dy=p(\omega )$ and $P(X_{2}=0)=q(\omega )=1-p(\omega )$, then the relationship between $\rho_{PB}=\text{cor}(Z_{1},Z_{2})$ (the biserial correlation) and $\rho_{B}=\text{cor}(Z_{1},X_{2})$ (the point‐biserial correlation) is due to Pearson (1909) and reported also in MacCallum et al. (2002), where the consequences of dichotomization for measurement and statistical analyses are illustrated and discussed in a more general context:

(1) $$\frac{\rho_{PB}}{\rho_{B}}=\frac{\varphi (\omega )}{\sqrt{pq}}.$$

**FIGURE 2:**
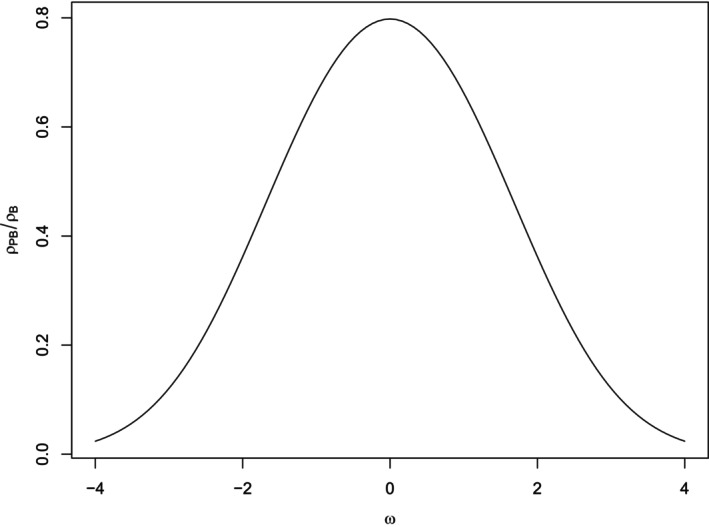
Maximal point‐biserial correlation (i.e., ratio between point‐biserial and biserial correlations) as a function of the cut‐point ω for a bivariate normal RV – Equation (1); the maximum, equal to 2φ(0), is attained at ω=0.

It is interesting to consider the plot of this function displayed in Figure 2 and to note that it is symmetrical and presents its unique maximum (equal to $2\varphi (0)=\sqrt{2/\pi }=.7979$) in ω=0, which corresponds to the ‘equal‐probability’ dichotomization (p=q=1/2). Note that changing the two values of the support of the discrete RV $X_{2}$, by default set at 0 and 1, as long as their order is preserved, does not affect the value of the biserial correlation coefficient (this is due to the well‐known invariance of Pearson's ρ under any positive linear transformation).

A generalization of Pearson's point‐biserial correlation to the case of discretization into a k point distribution, supported on {1,2,…,k}, is easily provided, again starting from a bivariate normal RV. Let, then, $X_{2}$ be the discrete RV obtained by discretizing the component $Z_{2}$. Recalling that the following relationship holds for the PDF of a standard normal RV:

∫xφ(x)dx=−φ(x)+constant,

it can be proved that the resulting Pearson's correlation coefficient between $Z_{1}$ and $X_{2}$, i.e., the point‐polyserial correlation coefficient, is

(2) $$\rho_{PP}=\text{cor}(Z_{1},X_{2})=\rho_{P}\overset{k}{\underset{i=1}{\sum }}\varphi \left[\Phi^{-1}(F_{i})\right]/\sqrt{\overset{k}{\underset{i=1}{\sum }}i^{2}p_{i}-{\left(\overset{k}{\underset{i=1}{\sum }}ip_{i}\right)}^{2}},$$

where $p_{i}$ and $F_{i}=\sum_{j=1}^{i}p_{j}$ are the probability and cumulative probability of the value i respectively, and $\rho_{P}=\text{cor}(Z_{1},Z_{2})$. Equation (2) indicates that there is a linear relationship between the polyserial and the point‐polyserial correlations, at least when working with a bivariate normal RV. The ratio between the point‐polyserial correlation and the (polyserial) correlation of the bivariate normal distribution is therefore constant once the $p_{i}$ are assigned and is equal to (see equation 12 in Olsson et al., 1982)

(3) $$\begin{array}{ll} \rho_{PP}/\rho_{P} & =\overset{k}{\underset{i=1}{\sum }}\varphi \left[\Phi^{-1}(F_{i})\right]/\sqrt{\overset{k}{\underset{i=1}{\sum }}i^{2}p_{i}-{\left(\overset{k}{\underset{i=1}{\sum }}ip_{i}\right)}^{2}}=\overset{k}{\underset{i=1}{\sum }}\varphi \left[\Phi^{-1}\left(\overset{i}{\underset{j=1}{\sum }}p_{j}\right)\right]/\sqrt{\overset{k}{\underset{i=1}{\sum }}i^{2}p_{i}-{\left(\overset{k}{\underset{i=1}{\sum }}ip_{i}\right)}^{2}},\; \end{array}$$

which consequently corresponds to the maximal point‐polyserial correlation, which is obtained by letting $\text{cor}(Z_{1},Z_{2})=1$.

We can particularize the formulas above in the case of discretization into k equal‐probability categories ($p_{i}=1/k$ for each i=1,…,k), i.e., if the discretized RV $X_{2}$ is defined as

$$X_{2}=\left\{\begin{array}{cc} 1\; & \text{if}\;Z_{2}<\Phi^{-1}(1/k),\\ i\; & \text{if}\;\Phi^{-1}\left(\frac{i-1}{k}\right)\leq Z_{2}<\Phi^{-1}\left(\frac{i}{k}\right),1<i<k,\\ k\; & \text{if}\;Z_{2}\geq \Phi^{-1}\left(\frac{k-1}{k}\right). \end{array}\right.$$

Then, specializing (3), we obtain

(4) $$\rho_{PP}/\rho_{P}=\overset{k-1}{\underset{i=1}{\sum }}\varphi (\Phi^{-1}(i/k))/\sqrt{(k^{2}-1)/12},$$

since $\varphi (\Phi^{-1}(1))=0$, and for a discrete uniform RV $X_{2}$, $\mathrm{34𝔼}(X_{2})=(k+1)/2$ and $\begin{array}{c} \mathrm{Var}(X_{2})=\sum_{i=1}^{k}i^{2}/k-{\left[(1+k)/2\right]}^{2}=(k+1)(2k+1)/6-{\left(k+1\right)}^{2}/4=(k^{2}-1)/12 \end{array}$.

## 4 MAXIMUM POINT‐POLYSERIAL CORRELATION UNDER NORMAL AND NON‐NORMAL DISTRIBUTIONS

If we consider a bivariate continuous RV $\left(Z_{1},Z_{2}\right)$ that is not bivariate normal, then (2) does not hold and one cannot claim there exists a linear relationship between the linear correlation coefficient before and after the discretization of $Z_{2}$. This means that for fixed k and $p_{i}$, the ratio between the correlations before and after discretization is not constant but depends on the value of the latter, although in some contributions, such as Bedrick (1995), Equation (2), and Demirtas and Vardar‐Acar (2017), an approximately linear relationship is presumed.

In the following subsections, we want to assess the maximum value that the point‐polyserial correlation can attain when we consider a RV X with an assigned continuous distribution, not necessarily normal, and a discrete RV $X_{d}$. We will review several continuous parametric families widely used in many fields of statistics, such as the uniform, exponential, Pareto, logistic, and power distributions. For each family we will derive the expression of the maximal point‐polyserial correlation as a function of the probabilities $p_{i}$ of the ordinalized distribution, and we will provide an algorithm that returns the maximum value of the maximal point‐polyserial correlation within the class of all possible k‐point distributions supported on {1,2,…,k}, discussing the features of the ordinal random distribution that produces this maximum value. We will also obtain analytically the limit of the maximal point‐polyserial correlation as k tends to ∞ when the ordinalized distribution is assumed to be uniform.

Then we will remove the assumption of CIS for the discretized RV and study the maximization problem, letting its support values themselves be variables along with their probabilities.

Note that the bivariate RV $\left(X,X_{d}\right)$ can be thought of as coming from the discretization of the second component of a bivariate continuous RV (X,Y). In this case, one can assume that Y has the same distribution as the unaltered continuous component X, but this is only required to let the polyserial correlation cor(X,Y) attain its natural upper bound +1: As recalled in Section 2, the maximum attainable correlation between two identically distributed RVs is always +1. In other words, computing the maximum point‐polyserial correlation between a continuous RV and ordinal/discrete RV does not strictly require specifying either the distribution of the latent continuous RV hypothetically underlying the latter or their joint continuous distribution. This would be required, however, if one needed to compute the point‐polyserial correlation given the value of the polyserial correlation.

### 4.1 General statement of problem

Let us consider an absolutely continuous RV X with known PDF f(x) and a discrete RV $X_{d}$ supported over k values $x_{1}<x_{2}<\text{⋯}<x_{k}$ with probabilities $p_{i}$ and cumulative probabilities $F_{i}$, i=1,…,k. The $p_{i}$ are unknown, and the $x_{i}$ can be assumed to be unknown quantities or can be fixed a priori to CIS. Let us assume that the first two moments of X exist and are μ=34𝔼(X) and $\sigma^{2}=\mathrm{Var}(X)$. $X_{d}$ may be thought of as the result of the discretization of a continuous RV with the same distribution as X. The objective is to find the maximum value of the linear correlation between X and $X_{d}$, $\text{cor}(X,X_{d})$, for a fixed k, by considering all the discrete distributions supported on k distinct values. The correlation can be written

(5) $$\rho_{PP}=\text{cor}(X,X_{d})=\frac{\mathrm{34𝔼}(XX_{d})-\mathrm{34𝔼}(X)\mathrm{34𝔼}(X_{d})}{\sqrt{\mathrm{Var}(X)}\sqrt{\mathrm{Var}(X_{d})}},$$

where $\mathrm{34𝔼}(X_{d})=\sum_{i=1}^{k}x_{i}p_{i}$ and $\mathrm{Var}(X_{d})=\sum_{i=1}^{k}x_{i}^{2}p_{i}-{\left(\mathrm{34𝔼}(X_{d})\right)}^{2}$. To compute the foregoing correlation, one would need to know the joint distribution of $\left(X,X_{d}\right)$, but to find its maximum value, this is not necessary. A first step is to recognize that this value will be taken when X and $X_{d}$ are comonotonic (Section 2). In this case, it is easy to see that the mixed moment, which we denote by ${\mathrm{34𝔼}}_{c}(XX_{d})$, where the subscript c stands for comonotonicity, can be written

(6) $${\mathrm{34𝔼}}_{c}(XX_{d})=\overset{k}{\underset{i=1}{\sum }}x_{i}\int_{F^{-1}(F_{i-1})}^{F^{-1}(F_{i})}xf(x)dx.$$

In the preceding formula, the values $F^{-1}(F_{i})$, for i=1,…,k−1, can be seen as thresholds induced on the continuous distribution of X by the distribution of $X_{d}$; the values $\int_{F^{-1}(F_{i-1})}^{F^{-1}(F_{i})}xf(x)dx/p_{i}$ are actually the conditional moments of X over the intervals $\left(F^{-1}(F_{i-1}),F^{-1}(F_{i})\right)$. Substituting (6) into (5) we obtain the expression of the point‐polyserial correlation in the case of comonotonicity between X and $X_{d}$, which we call the ‘maximal point‐polyserial correlation’; we will denote it by $\rho_{PP,\max}$. This expression depends on the $p_{i}$ and on the $x_{i}$, if these latter have not been assigned. One can then maximize (5), with the mixed moment expressed by (6), with respect to all the discrete distributions supported on k distinct values. If the $x_{i}$ are not fixed a priori, it can be shown (Bedrick, 1995) that their optimal values, given the probabilities $p_{i}$, are equal to

(7) $$x_{i}^{*}=\int_{F^{-1}(F_{i-1})}^{F^{-1}(F_{i})}xf(x)dx/p_{i}$$

or to a positive linear transformation thereof. It is well known that the $x_{i}^{*}$ in (7) preserve the expectation of X but underestimate its variance (Drezner & Zerom, 2016), i.e., for the resulting RV $X_{d}$, $\mathrm{34𝔼}(X_{d})=\mu$ and $\mathrm{Var}(X_{d})<\sigma^{2}$. We will refer to the $x_{i}^{*}$ as the ‘optimal scores’ (OPT), retaining the terminology in Bedrick (1995). With $x_{i}=x_{i}^{*}$, i=1,…,k, the correlation between $X_{d}$ and X, combining (5) with (6) and (7), can be rewritten as

(8) $$\text{cor}(X,X_{d})=\frac{\sum_{i=1}^{k}{x_{i}^{*}}^{2}p_{i}-\mu^{2}}{\sqrt{\left(\sum_{i=1}^{k}{x_{i}^{*}}^{2}p_{i}-\mu^{2}\right)\sigma^{2}}}=\frac{\sqrt{\sum_{i=1}^{k}{x_{i}^{*}}^{2}p_{i}-\mu^{2}}}{\sigma }=\sqrt{\frac{\mathrm{Var}(X_{d})}{\sigma^{2}}};$$

hence, maximizing the correlation between X and $X_{d}$ is equivalent to maximizing the variance of $X_{d}$ since $\sigma^{2}$ is fixed. But maximizing the point‐polyserial correlation (8) also with respect to the $p_{i}$ leads to the solution commonly referred to as the ‘optimal quantizer’ (Lloyd, 1982) or the set of ‘principal points’ (Flury, 1990). In fact, for the decomposition of the mean squared error (MSE) between X and the $x_{i}$ (see property (C) in theorem 1, Fang & Pan, 2023), we have that $\mathrm{Var}(X_{d})=\sigma^{2}-\text{MSE}(X_{d})$, so that maximizing $\mathrm{Var}(X_{d})$ is equivalent to minimizing the MSE between X and $X_{d}$. We will return to discussing quantization in Section 6.

Resuming, if we assume a CIS system for $X_{d}$, then the optimization problem can be stated as

(9) $$\begin{array}{lllll} & \underset{p_{1},\text{…},p_{k}}{\max}\; & \; & \text{cor}(X,X_{d})\; & \\ & \text{subject to}\; & & p_{i}\geq 0,\; & i=1,\text{…},k\\ & \; & & \overset{k}{\underset{i=1}{\sum }}p_{i}=1\; & \\ & \; & & x_{i}=i,\; & i=1,\text{…},k. \end{array}$$

If, instead, the support values of $X_{d}$ are unknown, then the optimization problem can be written

(10) $$\begin{array}{lllll} & \underset{p_{1},\text{…},p_{k};x_{1},\text{…},x_{k}}{\max}\; & \; & \text{cor}(X,X_{d})\; & \\ & \text{subject to}\; & & p_{i}\geq 0,\; & i=1,\text{…},k\\ & \; & & \overset{k}{\underset{i=1}{\sum }}p_{i}=1\; & \\ & \; & & x_{1}<x_{2}<\text{⋯}<x_{k}.\; & \end{array}$$

We will refer to Problems (9) and (10) as the ‘maximum point‐polyserial correlation problem’, with CIS and OPT support values respectively. Needless to say, the maximum value of the objective function, i.e., the maximum point‐polyserial correlation, will always be greater (or, at most, equal) for Problem (10). However, we would like to emphasize that assigning a CIS to the discrete variable $X_{d}$ in (9) is motivated by the fact that ordinal variables in real data sets may not provide any indication of the underlying continuous latent variable. Therefore, it is a fairly standard procedure to assign CIS to the ordered categories of $X_{d}$.

Although the two problems are generally not analytically solvable, we now provide an interesting general property of the solution to Problem (9).

Proposition 1 (Property of the solution to Problem (9).) *The solution of the optimization Problem* *(* 9 *)* *satisfies for all* k≥4 *the following equality*:

$$F^{-1}(F_{i+1})-F^{-1}(F_{i})=\text{constant},\;\text{for all}\;i=1,2,\text{…},k-2,$$

*where* $F_{i}=\sum_{j=1}^{i}p_{j}$, *and* $F^{-1}$ *is the quantile function of* X. *We can summarize this property by stating that the optimal cumulative probabilities* $F_{i}$ *mark on the continuous distribution of* X *equally spaced values*.

Let us start from the expression of the maximal point‐polyserial correlation in the case of CIS:

$$\rho_{PP,\max}=\frac{\overset{k}{\underset{i=1}{\sum }}i\int_{F^{-1}(F_{i-1})}^{F^{-1}(F_{i})}xf(x)dx-\mu \overset{k}{\underset{i=1}{\sum }}ip_{i}}{\sigma \sqrt{\overset{k}{\underset{i=1}{\sum }}i^{2}p_{i}-{\left(\overset{k}{\underset{i=1}{\sum }}ip_{i}\right)}^{2}}}.$$

We can rewrite Problem (9) as a non‐linear optimization problem by using Lagrange multipliers:

$$\begin{array}{ll} ℒ(p_{1},\text{…},p_{k},\lambda ) & =\frac{\sum_{i=1}^{k}i\int_{F^{-1}(F_{i-1})}^{F^{-1}(F_{i})}xf(x)dx-\mu \sum_{i=1}^{k}ip_{i}}{\sigma \sqrt{\sum_{i=1}^{k}i^{2}p_{i}-{\left(\sum_{i=1}^{k}ip_{i}\right)}^{2}}}+\lambda (\overset{k}{\underset{i=1}{\sum }}p_{i}-1).\; \end{array}$$

We can compute the partial derivative of the foregoing Lagrangian function with respect to $p_{j}$ and set it equal to zero, thereby obtaining

$$\begin{array}{ll} \frac{\partial ℒ(p_{1},\text{…},p_{k},\lambda )}{\partial p_{j}} & =\frac{\left[-\sum_{h=j}^{k-1}F^{-1}(F_{h})-j\mu ]\sigma \sqrt{V}-\frac{C\sigma }{2\sqrt{V}}(j^{2}-2jE\right)}{\sigma^{2}V}+\lambda \;\\ & =\frac{-\left[\sum_{h=j}^{k-1}F^{-1}(F_{h})+j\mu \right]V-C(j^{2}-2jE)/2}{\sigma V^{3/2}}+\lambda =0,\; \end{array}$$

where $E=\sum_{i=1}^{k}ip_{i}$ denotes the expectation of the discrete RV, $V=\sum_{i=1}^{k}i^{2}p_{i}-{\left(\sum_{i=1}^{k}ip_{i}\right)}^{2}$ its variance, and C its covariance with X; in the notation, for the sake of simplicity we omitted the dependence on the $p_{i}$. Since for the optimal solution the foregoing equation must be satisfied for any feasible value of j, by evaluating it for two consecutive values of j, we obtain

$$\begin{array}{ll} -\left[\overset{k-1}{\underset{h=j}{\sum }}F^{-1}(F_{h})+j\mu \right]V-C(j^{2}-2jE)/2+\lambda \sigma V^{3/2} & =0\;\\ -\left[\overset{k-1}{\underset{h=j+1}{\sum }}F^{-1}(F_{h})+(j+1)\mu \right]V-C({\left(j+1\right)}^{2}-2(j+1)E)/2+\lambda \sigma V^{3/2} & =0.\; \end{array}$$

By subtracting the former from the latter, we obtain

$$\begin{array}{l} \left[F^{-1}(F_{j})-\mu \right]V-C(j+1/2-E)=0, \end{array}$$

and again, by considering two consecutive values for the index j, we can derive that for the optimal solution to (9), for all i=1,…,k−2, the following equality holds:

$$F^{-1}(F_{i+1})-F^{-1}(F_{i})=C/V.$$

Since the covariance C can be written $C=\rho_{PP}^{*}\sigma \sqrt{V}$, the preceding result can be restated as

$$F^{-1}(F_{i+1})-F^{-1}(F_{i})=\rho_{PP}^{*}\sigma /\sqrt{V},$$

where $\rho_{PP}^{*}=\max\rho_{PP,\max}$ represents the maximum correlation value attained.

Thanks to this result, it is possible to supply an alternative equivalent formulation of Problem (9).

Proposition 2 (Alternative statement of Problem (9).) *Problem* (9) *can be restated as follows*: 
(11)

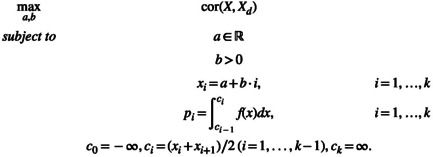

This formulation is simpler, since now there are just two variables on which to optimize the objective function: a shift variable a and a scale variable b>0, which define the equally spaced support points $x_{i}$ as a positive linear transformation of the CIS. However, to define the probabilities, one must introduce the thresholds $c_{i}$, built as midpoints between consecutive support values, which are equally spaced values as well. The optimal solution to Problem (11) (i.e., the optimal values of a and b) will yield the same $p_{i}$ and the same value of maximum correlation as (9); the optimal support values $x_{1},\text{…},x_{k}$ will be generally different from 1,…,k.

### 4.2 Normal

**FIGURE 3:**
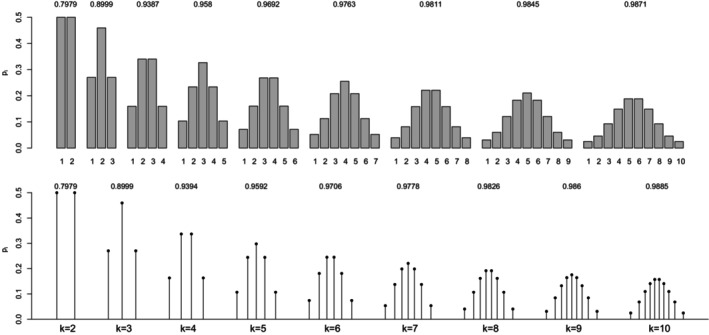
Maximal point‐polyserial correlations and corresponding configurations $p_{1},\text{…},p_{k}$ for a number of categories k=2,…,10 when the continuous distribution is normal. In the top panel, we consider CISs for the ordered categories; in the bottom panel, the support values (OPT) are determined along with the probabilities as a solution to the optimization problem.

For a (standard) normal RV X, the maximal correlation with an ordinal k‐point RV $X_{d}$ equals the ratio in (3). Figure 3 displays for k=2,…,10 the optimal solution to Problems (9) (top panel) and (10) (bottom panel). For each k, a bar plot (for the CIS) and a stick plot (for OPT) are drawn that represent the k optimal probabilites $p_{i}$ leading to the maximum point‐polyserial correlation. For OPT, the optimal support values $x_{i}$ are displayed considering the scale of the x‐axis. Starting from the CIS, we notice that all these ordinal distributions maximizing the maximal point‐polyserial correlation are symmetrical, as one could have expected, with a unique mode – the central category – if k is odd, and with two modes – the central categories – if k is even. Therefore, at least when k is odd, they inherit or, better, mirror the two main features of the continuous Gaussian distribution, symmetry and unimodality. The results for OPT are similar; the optimal distribution shows for the same k≥4 slightly different probabilities and support values that are slightly unequally spaced. The increase in the maximum correlation is quite negligible. For illustrative purposes, we report here the R code used to determine the value of the maximal point‐polyserial correlation, Problem (9), for k=5.

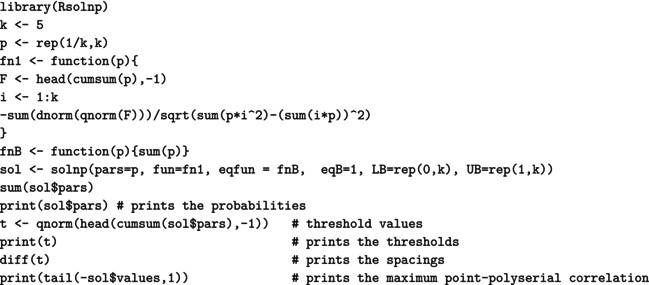

which produces the following output:

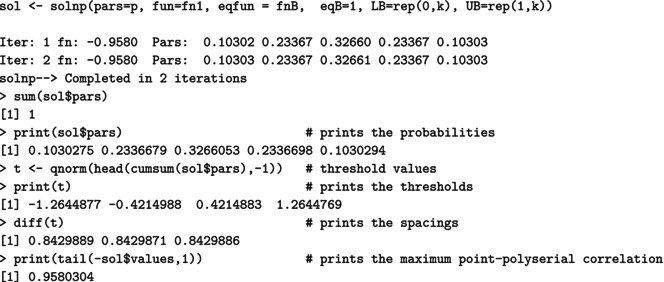

The R code used to solve Problem (10), with k=5, is as follows:

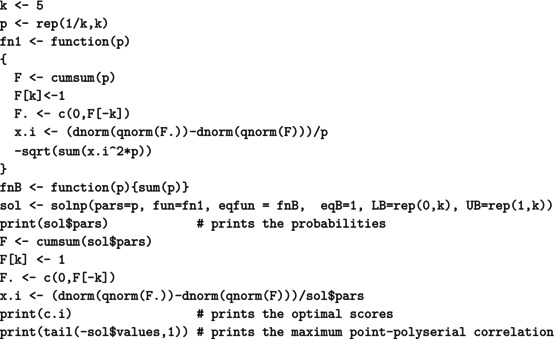

which produces the output:

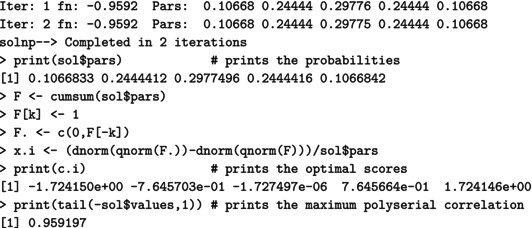

We used the solnp function included in the Rsolnp package (Ghalanos & Theussl, 2015; Ye, 1987) to solve the non‐linear maximization problems, which are actually converted into minimization problems by simply changing the sign to the expression of the maximal point‐polyserial correlation for an underlying normal distribution (3). The constraints on the $p_{i}$ are provided through the arguments eqfun and eqB (through which we impose that $\sum_{i=1}^{k}p_{i}=1$), LB (lower bounds for the $p_{i}$), and UB (upper bounds for the $p_{i}$). For Problem (10), the optimal scores are obtained recalling (7), adapted to the standard normal distribution, and saved in the R object x.i.

From the R output, one can check the feature of the optimal $p_{i}$ for Problem (9), expressed by Proposition (1): The corresponding thresholds (i.e., the quantiles of level $F_{i}$ of the continuous RV) constitute a set of equally spaced values. It should also be expected that the k‐point distribution maximizing the point‐polyserial correlation will be symmetrical, i.e., $p_{j}=p_{k+1-j}$, j=1,…,k; hence, the thresholds are symmetrical around zero.

If we consider a discrete uniform RV $X_{d}$, then the maximal point‐polyserial correlation is equal to the ratio in (4). For k→∞, it tends asymptotically to $\sqrt{3/\pi }$. In fact, we can write 
(12)

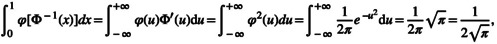

but the integral on the left‐hand side of (12) is related to the finite sum above through

$$\begin{array}{c} \underset{k\to \infty }{\lim}\frac{1}{k}\underset{i=1}{\sum }k\varphi [\Phi^{-1}(i/k)]=\underset{k\to \infty }{\lim}\frac{1}{k}\overset{k-1}{\underset{i=1}{\sum }}\varphi [\Phi^{-1}(i/k)]=\int_{0}^{1}\varphi [\Phi^{-1}(x)]dx=\frac{1}{2\sqrt{\pi }}, \end{array}$$

and then it easily follows that

$$\underset{k\to \infty }{\lim}\rho_{PP,\max}=\frac{2\sqrt{3}}{2\sqrt{\pi }}=\sqrt{3/\pi }\approx 0.977205.$$

This is an interesting theoretical result: Starting from a bivariate standard normal distribution with correlation coefficient ρ and discretizing one of its components through an equal‐probability discretization process, the resulting correlation coefficient between the unaltered component and the new discrete one, letting k go to ∞, tends to a value strictly smaller than ρ. This result is not unexpected since discretizing a normal distribution, although through ‘many’ equal‐probability categories, produces a distribution that cannot resemble the unimodal normal PDF (see, e.g., Barbiero & Hitaj, 2023; Table 1).

**TABLE 1:** Maximal point‐polyserial correlation as a function of k, number of equal‐probability categories, for a normal distribution.

k	2	3	4	5	6	7	8	9	10	20	50	100	1000
$\rho_{PP}/\rho$	.7979	.8906	.9253	.9423	.9520	.9581	.9622	.9650	.9672	.9744	.9767	.9771	.9772

We concisely summarize the main results concerning the maximum point‐polyserial correlation for the normal distribution in the following proposition.

Proposition 3 (Normal distribution.) *For the standard normal distribution, the optimal solution to Problem* (9) *has symmetric probabilities*, $p_{j}=p_{k+1-j},j=1,\text{…},k$, *with a unique mode in* (k+1)/2 *if* k *is odd, two modes in* k/2 *and* k/2+1 *if* k *is even. For the same* k, *the optimal solution to problem* (10) *has a slightly different symmetric distribution (with the same features as for the CIS) with unequally spaced support values; the maximum value of correlation is just barely larger than for Problem* (9).

### 4.3 Uniform

Let X be a uniform RV in (0,1), with f(x)=1,x∈[0,1]; then 34𝔼(X)=1/2 and Var(X)=1/12. Then the mixed moment in the case of comonotonicity between X and $X_{d}$ becomes

$${\mathrm{34𝔼}}_{c}(XX_{d})=1\cdot \int_{0}^{F_{1}}xdx+2\cdot \int_{F_{1}}^{F_{2}}xdx+\text{⋯}+k\cdot \int_{F_{k-1}}^{1}xdx=\overset{k}{\underset{i=1}{\sum }}i\cdot \frac{F_{i}^{2}-F_{i-1}^{2}}{2}=\frac{1}{2}\left(k-\overset{k-1}{\underset{i=1}{\sum }}F_{i}^{2}\right).$$

Problem (9) can be rewritten, following the lines of Proposition (1), as

$$\begin{array}{l} \max_{p_{1},\text{…},p_{k}}\left[\frac{1}{2}\left(k-\sum_{i=1}^{k-1}F_{i}^{2}\right)-\frac{1}{2}\sum_{i=1}^{k}ip_{i}\right]/\sqrt{\frac{1}{12}\left(\sum_{i=1}^{k}i^{2}p_{i}-{\left(\sum_{i=1}^{k}ip_{i}\right)}^{2}\right)}, \end{array}$$

subject to the usual constraints on the $p_{i}$. Using the notation V for the variance of $X_{d}$, with E its expectation, and with C the covariance between X and $X_{d}$ (E, V, and C all depend on the $p_{i}$, but for the sake of simplicity we omitted this dependence in the notation), the equation obtained by setting the partial derivative of the Lagrangian function with respect to $p_{i}$ equal to zero is

$$\frac{1}{2}\left[-\left(2\overset{k-1}{\underset{j=i}{\sum }}(k-j+1)p_{j}+i\right)V-0.5\left(k-\overset{k-1}{\underset{i=1}{\sum }}{\left(\overset{i}{\underset{j=1}{\sum }}p_{i}\right)}^{2}-\sum ip_{i}\right)(i^{2}-2i\overset{k}{\underset{i=1}{\sum }}ip_{i})\right]/V^{3/2}+\lambda =0,i=1,\text{…},k-1.$$

Subtracting the second (i=2) from the first (i=1) equation we obtain

$$-\frac{1}{2}(1-2p_{1})V-\frac{1}{2}C(3-2E)=0,$$

and then

$$(1-2p_{1})V+C(3-2E)=0,$$

from which

$$p_{1}=\frac{V-C(2E-3)}{2V}=\frac{1}{2}-\frac{C(2E-3)}{2V}.$$

Subtracting the third and the second equation yields

$$-\frac{1}{2}(1-2p_{1}-2p_{2})V-\frac{1}{2}C(5-2E)=0,$$

from which, recalling the previous expression obtained for $p_{1}$,

$$\left[1-1+\frac{C}{V}(2E-3)-2p_{2}\right]V+C(5-2E)=0,$$

from which one derives $p_{2}=C/V$ and, in a similar manner, $p_{3}=\text{⋯}=p_{k-1}=C/V$; finally, $p_{k}=\frac{1}{2}-\frac{C}{2V}(2k-1-2E)$.

Although the preceding optimization problem cannot be solved analytically in a direct way, it can be proved that for any k≥2, the discrete uniform distribution, which assigns each value i∈{1,2,…,k} a constant probability $p_{i}=1/k$, is the one, among all the k‐point discrete distributions sitting on {1,2,…,k}, that maximizes the maximal point‐polyserial correlation. In fact, letting $p_{i}=1/k$ for all i=1,…,k, we obtain E=(k+1)/2, $V=(k^{2}-1)/12$, $\begin{array}{c} C=𝔼(XX_{d})-𝔼(X)𝔼(X_{d})=\frac{1}{2}\left[k-\sum_{i=1}^{k-1}{\left(i/k\right)}^{2}\right] \end{array}$ $\begin{array}{c} -(k+1)/4=\frac{1}{2}\left[k-\frac{1}{k^{2}}\frac{\left(k-1)k(2(k-1)+1\right)}{6}\right]-(k+1)/4=(k^{2}-1)/(12k) \end{array}$, and all the equations obtained by setting equal to zero the derivatives of the Lagrangian function are satisfied. What follows is the R code that can be used to numerically determine the solution to (9) with a number of categories from 2 to 10:

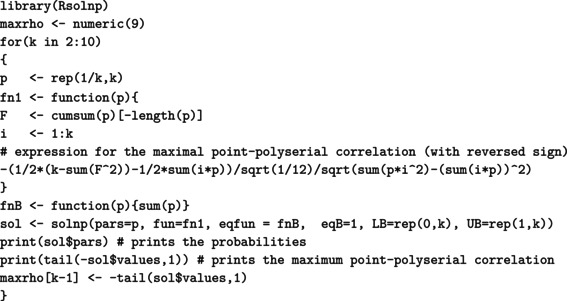

Table 2 displays for several values of k the maximum point‐polyserial correlation, for which an analytic expression is readily obtained:

$$\max\rho_{PP,\max}=\frac{\frac{k^{2}-1}{12k}}{\sqrt{\frac{k^{2}-1}{12}\frac{1}{12}}}=\frac{\sqrt{k^{2}-1}}{k}=\sqrt{1-1/k^{2}}.$$

We thus observe that $\lim_{k\to \infty }\rho_{PP,\max}=1$. As the number of categories k increases, the maximum point‐polyserial correlation approaches 1, which is the natural upper bound of Pearson's correlation.

**TABLE 2:** Values of maximal point‐polyserial correlation between a RV uniformly distributed in [0,1] and a discrete RV for several values of k.

k	2	3	4	5	6	7	8	9	10	20	50	100	200
$\max\rho_{PP,\max}$	.8660	.9428	.9682	.9798	.9860	.9897	.9922	.9938	.9950	.9987	.9998	.99995	≈1.0000

If we remove the hypothesis of CIS for the ordinal RV, the results about the maximal point‐polyserial correlation would not change: The OPT would be equally spaced values in (0,1), $x_{i}^{*}=(2i-1)/(2k)$, i=1,…,k (Zoppè, 1995) and, thus, turn out to be positive linear transforms of the CIS. We concisely summarize these main results by the following proposition.

Proposition 4 (Uniform distribution.) *For a standard uniform distribution, the optimal solution for both Problems* (9) *and* (10) *has constant probabilities* $p_{i}=1/k$, i=1,…,k. *For the latter, the optimal support values are equally spaced*: $x_{i}=(2i-1)/(2k)$. *The maximum value of the correlation in both cases is* $\sqrt{1-1/k^{2}}$.

It is important to note that, although it is quite easy to derive the expression of the maximal point‐polyserial correlation, starting from the (bivariate) continuous distribution, finding the point‐polyserial correlation and then the correlation ratio is more challenging or, more specifically, it requires some additional information: While for the former it is sufficient to fully specify the univariate non‐normal continuous distribution, for the latter it is necessary to specify the joint random distribution of $\left(Z_{1},Z_{2}\right)$ or, equivalently, the two marginal distributions of $Z_{1}$ and $Z_{2}$ and the copula $C(u_{1},u_{2})$ linking them into the joint distribution. To better understand this point, we carried out the following numerical experiment. We considered four different parametric copulas $C(u_{1},u_{2};\theta )$ (Gauss, Frank, Clayton, and Gumbel), whose marginal distributions are by definition standard uniform. For each copula and for different values of the linear correlation ρ (the biserial/polyserial correlation), properly induced by the copula parameter θ, we computed the point‐biserial/polyserial correlation, and the corresponding ratio, by considering for the sake of simplicity k=2 and k=3 equal‐probability categories (which are assigned CISs) for the discretized random variable. The results indicate that the ratio between point‐polyserial and polyserial correlations is not constant with ρ (although it can be treated as nearly constant), confirming the fact that a constant ratio characterizes the (bivariate) normal distribution only (Equations 2 and 3). The range of values that the ratio can span, though narrow, sensibly varies depending on the copula selected. We considered only positive values of ρ since, whereas the Frank and Gauss copulas are comprehensive (i.e., they are able to model the entire range of dependence, from countermonotonicity to comonotonicity, passing through independence), and then they are able to induce all the values of ρ in [−1,+1]), the Gumbel and Clayton copulas can only model positive dependence and, thus, induce only positive values of linear correlation. The point‐biserial (point‐polyserial) correlation can be computed as usual as

$$\begin{array}{l} \rho_{PP}=\frac{\mathrm{34𝔼}(U_{1}U_{2d})-\mathrm{34𝔼}(U_{1})\mathrm{34𝔼}(U_{2d})}{\sqrt{\mathrm{Var}(U_{1})\mathrm{Var}(U_{2d})}}, \end{array}$$

where the value of the mixed moment can be expressed, in the case of two equal‐probability categories for $U_{2d}$, as

(13) $$\begin{array}{l} \mathrm{34𝔼}(U_{1}U_{2d})=1\int_{0}^{1}du_{1}\int_{0}^{1/2}u_{1}c(u_{1},u_{2};\theta )du_{2}+2\int_{0}^{1}du_{1}\int_{1/2}^{1}u_{1}c(u_{1},u_{2};\theta )du_{2}, \end{array}$$

where $c(u_{1},u_{2};\theta )$ is the copula density, with $\mathrm{34𝔼}(U_{1})=1/2$, $\mathrm{34𝔼}(U_{2d})=3/2$, $\mathrm{Var}(U_{1})=1/12$, $\mathrm{Var}(U_{2d})=1/4$. In the case of three equal‐probability categories for $U_{2d}$, the value of the mixed moment takes on the expression

(14) $$\begin{array}{l} \mathrm{34𝔼}(U_{1}U_{2d})=1\int_{0}^{1}du_{1}\int_{0}^{1/3}u_{1}c(u_{1},u_{2};\theta )du_{2}+2\int_{0}^{1}du_{1}\int_{1/3}^{2/3}u_{1}c(u_{1},u_{2};\theta )du_{2}+3\int_{0}^{1}du_{1}\int_{2/3}^{1}u_{1}c(u_{1},u_{2};\theta )du_{2}, \end{array}$$

and it is easy to check that now $\mathrm{34𝔼}(U_{2d})=2$ and $\mathrm{Var}(U_{2d})=2/3$. The point‐polyserial correlation is readily computed once the quantities in (13) and (14) are evaluated: To this end, one can resort to the cubature package (Narasimhan et al., 2023) in R, which implements adaptive multivariate integration over hypercubes. The function iRho, provided by the package copula (Hofert et al., 2023), determines (“calibrates”) the copula parameter θ given the value of Spearman's rank correlation, which coincides with Pearson's correlation for a bivariate copula.

**FIGURE 4:**
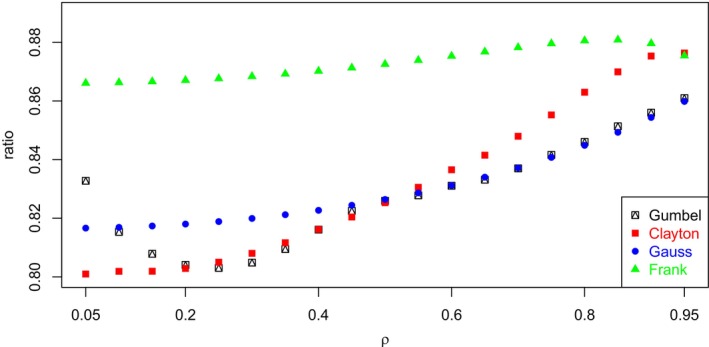
Graph of ratio between point‐biserial correlation and biserial correlation for several copulas and values of ρ; we assumed $p_{1}=p_{2}=1/2$ for the dichotomous RV.

**FIGURE 5:**
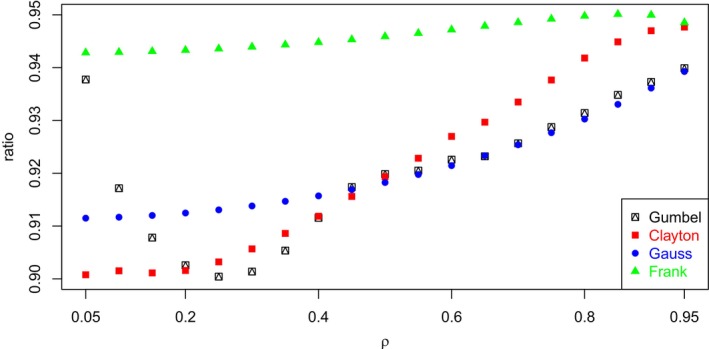
Graph of ratio between point‐polyserial (k=3) correlation and biserial correlation for several copulas and values of ρ; we assumed $p_{1}=p_{2}=p_{3}=1/3$ for the ordinal RV.

Figure 4 displays, for each copula examined, the values of the ratio between point‐biserial and biserial correlations for different values of the latter (from .05 to .95 in steps of .05). Note that the values of the ratio all cluster around the value .8660, which is reported in Table 2 as the maximum value of point‐biserial correlation for a uniform distribution. Analogously, Figure 5 displays, for each copula examined, the values of the ratio between point‐polyserial and polyserial (k=3) correlations for different values of the latter (the same grid as was adopted for k=2). Note the values of the ratio all cluster around the value .9428, which is reported in Table 2 as the maximum value of point‐biserial correlation for the uniform distribution for k=3.

### 4.4 Exponential

Let X be an exponential RV with PDF $f(x)=\lambda e^{\;-\;\lambda x}$ and cumulative distribution function (CDF) $F(x)=1-e^{\;-\;\lambda x}$, x>0, λ>0. It is well known that 34𝔼(X)=1/λ and $\mathrm{Var}(X)=1/\lambda^{2}$. The quantile of level 0<u<1 is $x_{u}=-\log(1-u)/\lambda$.

The mixed moment between X and $X_{d}$ when they are comonotonic and CISs are used for the latter RV is obtained by recalling (6)

$$\begin{array}{cc} {𝔼}_{c}(XX_{d}) & =\overset{k}{\underset{i=1}{\sum }}i\int_{F^{-1}(F_{i-1})}^{F^{-1}(F_{i})}x\lambda e^{\;-\;\lambda x}dx=\overset{k}{\underset{i=1}{\sum }}i{\left[-(x+1/\lambda )e^{\;-\;\lambda x}\right]}_{-\log(1-F_{i-1})/\lambda }^{-\log(1-F_{i})/\lambda }\\ & =\frac{1}{\lambda }\overset{k}{\underset{i=1}{\sum }}i\{[\log(1-F_{i})-1](1-F_{i})-[\log(1-F_{i-1})-1](1-F_{i-1})\}=\frac{1}{\lambda }\left[1-\overset{k-1}{\underset{i=1}{\sum }}[\log(1-F_{i})-1](1-F_{i})\right]\; \end{array}$$

since

$$\int_{a}^{b}x\lambda e^{\;-\;\lambda x}dx={\left[-\left(x+\frac{1}{\lambda }\right)e^{\;-\;\lambda x}\right]}_{a}^{b}.$$

Therefore, the expression of the corresponding maximal point‐polyserial correlation is

(15) $$\begin{array}{l} \rho_{PP,\max}=\frac{1-\sum_{i=1}^{k-1}[\log(1-F_{i})-1](1-F_{i})-\sum_{i=1}^{k}ip_{i}}{\sqrt{\sum_{i=1}^{k}i^{2}p_{i}-{\left(\sum_{i=1}^{k}ip_{i}\right)}^{2}}}. \end{array}$$

**FIGURE 6:**
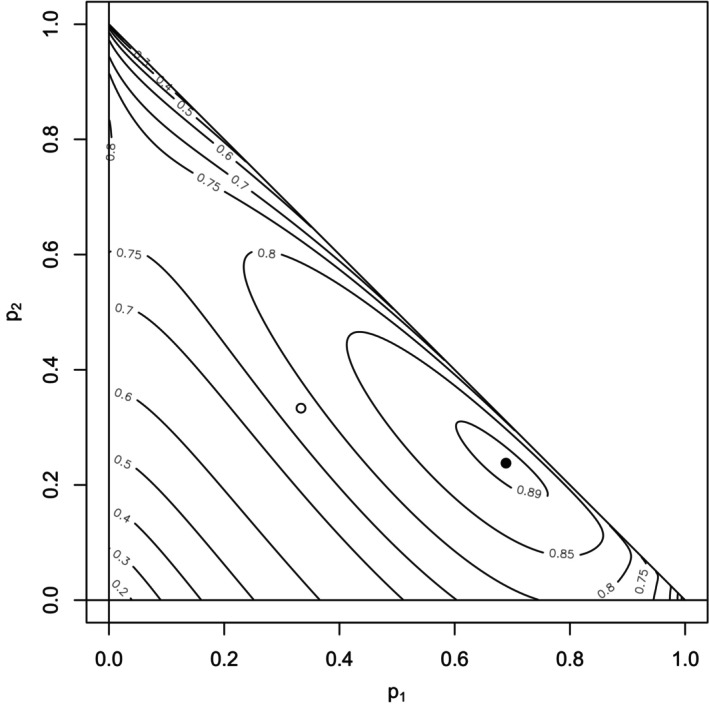
Level curves for maximal point‐polyserial correlation (15) between an exponential distribution and a discrete RV with k=3 ordered categories, which are assigned CISs, with probabilities $p_{1}$, $p_{2}$, and $p_{3}$. The point of the coordinates (1/3,1/3), corresponding to the discrete uniform distribution, is represented by the empty circle between the contour lines of levels .75 and .8. The pair $\left(p_{1},p_{2}\right)$ maximizing (15) is represented by the filled circle inside the contour line of level .89 (refer also to Figure 7, top panel, second bar plot from left).

As an example, Figure 6 displays the level curves of the maximal point‐polyserial correlation in (15) for k=3 as a function of the probabilities $p_{1}$ and $p_{2}$ (which must satisfy $0\leq p_{1}+p_{2}\leq 1$), since $p_{3}=1-p_{1}-p_{2}$. Figure 6 can be seen as the trivariate analogue of figure 1 in (Cheng & Liu, 2016) for the exponential distribution.

**FIGURE 7:**
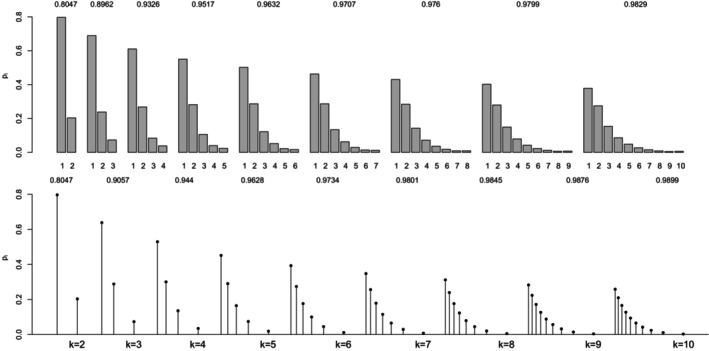
Solution to maximal point‐polyserial correlation problem for exponential distribution for different values of k. In the top panel, we consider CISs for the ordered categories; in the bottom panel, the support values (OPT) are determined along with the probabilities as a solution to the optimization problem.

Maximizing the function in Equation (15), for a fixed k, with respect to the $p_{i}$ (i.e., solving Problem (9)) does not return a closed‐form solution; one must resort to numerical optimization as was already done for the normal and uniform distributions. The k‐point distribution maximizing the maximal point‐polyserial correlation is empirically proved to have decreasing probabilities $p_{i}$ for k≤7, thereby resembling the trend of the exponential PDF; for k≥8 the probabilities are decreasing till the second to last category, but the last category has a larger, though very small, probability than the former ($p_{k}>p_{k-1}$); one can empirically ascertain this by looking at the three right‐most graphs of Figure 7 (top panel), where the k‐point discrete distributions maximizing $\rho_{PP,\max}$ are displayed for k=2,3,…,10 (top panel). We note that, for any k there examined, the values of $\rho_{PP,\max}$ for the exponential distribution are not very different from the analogue values for the normal distribution, reported in Figure 3, and are a bit smaller than those obtained for the uniform distribution. Despite being strongly asymmetrical, the exponential distribution is still able to assure high values of a point‐polyserial correlation.

If we restrict our attention to a uniform discrete RV, then $F_{i}=i/k$, and the i/k‐order quantile is $x_{i/k}=\frac{\log k-\log(k-i)}{\lambda },$and then one obtains, by specializing Equation (15), after some algebraic steps, the following expression for the maximum mixed moment:

$$\begin{array}{ll} \max\mathrm{34𝔼}{\left(XX_{d}\right)}^{\text{(eq)}} & =\frac{1}{\lambda k}\left[\frac{k(k+1)}{2}(1+\log k)-\overset{k}{\underset{i=2}{\sum }}i\log i\right]\; \end{array}$$

and for the maximal point‐polyserial correlation:

$$\begin{array}{ll} \rho_{PP,\max}^{\text{(eq)}} & =\frac{\frac{1}{k}\left[k(k+1)(1+\log k)/2-\sum_{i=2}^{k}i\log i\right]-\frac{k+1}{2}}{\sqrt{(k^{2}-1)/12}},\; \end{array}$$

which tends to $\sqrt{3}/2\approx 0.866$ as k tends to infinity. In fact, since

$$\int_{1}^{k}x\log xdx={\left[\frac{1}{4}x^{2}(2\log x-1)\right]}_{1}^{k}=\frac{1}{2}k^{2}\log k-\frac{1}{4}(k^{2}-1)$$

and the sum appearing in the numerator of $\rho_{PP,\max}^{\text{(eq)}}$ can be approximated for large k as

$$\overset{k}{\underset{i=2}{\sum }}i\log i=\overset{k}{\underset{i=1}{\sum }}i\log i\approx \frac{1}{2}k^{2}\log k-\frac{1}{4}(k^{2}-1),$$

it is immediate to prove the asymptotic result.

Table 3 reports the values of the maximal point‐polyserial correlation under the equal‐probability setting for different values of k. By comparing them to the values of the maximum point‐polyserial correlations displayed in Figure 7 (top panel), we can conclude that properly diversifying the probabilities of the k categories significantly increases the maximal value of point‐polyserial correlation even when k becomes larger: For k=10, the increase in the maximal correlation is approximately 15%, and this is ascribable to the highly non‐uniform and asymmetrical nature of the exponential PDF. Moreover, note that the limiting value of the point‐polyserial correlation for the exponential distribution under the equal‐probability setting is quite a bit smaller than its analogue resulting for the normal RV ($\sqrt{3}/2<\sqrt{3/\pi }$); this clearly derives from the asymmetrical nature of the exponential distribution, which mismatches with the equal probabilities characterizing the k‐point discrete uniform RV considered in the limit case.

**TABLE 3:** Maximal point‐polyserial correlation between an exponentially distributed RV and an ordinal RV with k equal‐probability categories.

k	2	3	4	5	6	7	8	9	10	20	50	100	1000
$\rho_{max}^{PP}$	.6931	.7796	.8130	.8297	.8395	.8456	.8498	.8528	.8550	.8628	.8654	.8658	.8660

If we consider the OPT instead of the CIS, then the k‐point distributions maximizing the point‐polyserial correlation are, for each k, different. They are characterized by decreasing probabilities $p_{i}$, by support values that now depend on λ, and increasing spacings between consecutive support values. We recall that for the optimal k‐point distribution (which coincides with the optimal quantizer) the jth spacing is equal to the (j+1)th spacing of the optimal (k+1)‐point distribution, i.e., the series of spacings repeats itself (Zoppè, 1995). The maximum correlation for k≥3 is larger than in the case of the CIS. These results are graphically displayed in Figure 7 (bottom panel), where the parameter λ is set equal to 1. Indeed, changing the parameter value from $\lambda_{1}$ to $\lambda_{2}$ simply translates into a scale transformation with a factor $\lambda_{1}/\lambda_{2}$ for the optimal support values.

We concisely summarize the main results for the exponential distribution in the following proposition.

Proposition 5 (Exponential distribution.) *The optimal solution to Problem* (9) *has decreasing* $p_{i}$ *for* k≤7. *The optimal solution to Problem* (10), *for the same* k≥3, *has decreasing* $p_{i}$ *and yields a slightly larger maximum value of correlation. We observe that compared to CIS, smaller support values are assigned smaller probabilities and larger support values are assigned larger probabilities*.

### 4.5 Pareto (Lomax)

The one‐parameter Pareto distribution is characterized by the PDF $f(x)=\alpha /x^{\alpha +1}$ and the CDF $F(x)=1-1/x^{\alpha }$ for x>1, with α>0; its expectation is 34𝔼(X)=α/(α−1) for α>1; its variance is $\mathrm{Var}(X)=\alpha /[{\left(\alpha -1\right)}^{2}(\alpha -2)]$ for α>2. The quantile function is $x_{u}=F^{-1}(u)=1/{\left(1-u\right)}^{1/\alpha }$, 0<u<1.

It is easy to find the expression of the mixed moment when the two RVs X and $X_{d}$ are comonotonic and the ordered categories of $X_{d}$ are assigned CISs; it is equal to

(16) $$\begin{array}{l} {\mathrm{34𝔼}}_{c}(XX_{d})=\overset{k}{\underset{i=1}{\sum }}i\int_{F^{-1}(F_{i-1})}^{F^{-1}(F_{i})}\frac{\alpha x}{x^{\alpha +1}}dx=\overset{k}{\underset{i=1}{\sum }}i\frac{\alpha }{1-\alpha }{\left[x^{1-\alpha }\right]}_{1/{\left(1-F_{i-1}\right)}^{1/\alpha }}^{1/{\left(1-F_{i}\right)}^{1/\alpha }}=\frac{\alpha }{\alpha -1}\left[1+\overset{k-1}{\underset{i=1}{\sum }}\frac{1}{{\left(1-F_{i}\right)}^{\frac{1-\alpha }{\alpha }}}\right]. \end{array}$$

The corresponding maximal point‐polyserial correlation can then be determined; its maximum value, for a given k, can be obtained solving the optimization Problem (9) numerically. Here, in Table 4, we report the maximum value of the point‐polyserial correlation for several combinations of the Pareto parameter α and of the number of categories k.

**TABLE 4:** Maximum point‐polyserial correlation between a Pareto‐distributed RV with parameter α and a discrete RV with k categories.

α, k	2	3	4	5	6	7	8	9	10	20
3	.6813	.7716	.8148	.8413	.8596	.8731	.8837	.8922	.8992	.9350
4	.7345	.8274	.8695	.8942	.9106	.9224	.9313	.9382	.9439	.9700
5	.7556	.8488	.8899	.9134	.9286	.9393	.9473	.9534	.9583	.9800

**FIGURE 8:**
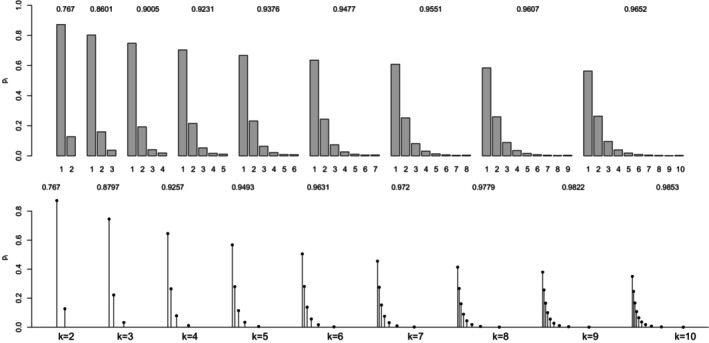
Solution to maximal point‐polyserial correlation problem for a Pareto distribution with parameter α=6 for different values of k. In the top panel, we consider the CIS for the ordered categories; in the bottom panel, the support values (OPT) are determined along with the probabilities as a solution to the optimization problem.

Figure 8 displays, for k=2,…,10, the discrete distribution solutions to the maximal point‐polyserial correlation problem when α=6, for CIS (top panel) and OPT (bottom panel).

In general, focusing on the CIS, for an assigned k, it can be shown numerically that the discrete distribution maximizing the point‐polyserial correlation has most of the probability concentrated in the first category, whereas much smaller probabilities are assigned to the others. Furthermore, we observe that for each k, $p_{1}>p_{2}>\text{⋯}>p_{k-1}$. This behaviour is similar to that of the exponential distribution; we note that for the same number of categories k, the maximum value of the point‐polyserial correlation for the Pareto distribution is smaller for any value of α than for the exponential distribution.

Focusing on the equal‐probability case, since the expression of the quantile of level i/k is $x_{i/k}={\left(\frac{k}{k-i}\right)}^{1/\alpha }$, it is easy to compute the mixed moment arising when the two RVs are comonotonic, specializing the general expression in (16):

$${\mathrm{34𝔼}}_{c}{\left(XX_{d}\right)}^{\text{(eq)}}=\overset{k}{\underset{i=1}{\sum }}i\int_{x_{(i-1)/k}}^{x_{i/k}}x\cdot \frac{\alpha }{x^{\alpha +1}}dx=\overset{k}{\underset{i=1}{\sum }}i{\left[\frac{\alpha }{1-\alpha }x^{1-\alpha }\right]}_{{\left(\frac{k}{k-i}\right)}^{1/\alpha }}^{{\left(\frac{k}{k-i+1}\right)}^{1/\alpha }}=\frac{\alpha }{\alpha -1}\left[1+\overset{k-1}{\underset{i=1}{\sum }}{\left(\frac{k}{k-i}\right)}^{(1-\alpha )/\alpha }\right],$$

and therefore the expression of the maximum point‐polyserial correlation becomes

$$\begin{array}{ll} \rho_{PP,\max}^{\text{(eq)}} & =\frac{\frac{\alpha }{\alpha -1}\left[1+\sum_{i=1}^{k-1}{\left(\frac{k}{k-i}\right)}^{(1-\alpha )/\alpha }-(k+1)/2\right]}{\sqrt{\frac{\alpha }{{\left(\alpha -1\right)}^{2}(\alpha -2)}\frac{k^{2}-1}{12}}}.\; \end{array}$$

Since we have that 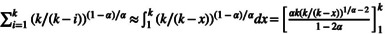 
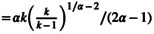
, then for k→∞, provided that α>2,

$$\underset{k\to \infty }{\lim}\rho_{PP,\max}^{\text{(eq)}}=\frac{\sqrt{3\alpha (\alpha -2)}}{2\alpha -1}.$$

For α=3 we have $\rho_{pp,\max}^{\text{(eq)}}=0.6$; for α=5 we have $\rho_{pp,\max}^{\text{(eq)}}=0.745356$; for α→∞, the maximum of the point‐polyserial correlation, under the equal‐probability setting, tends to $\sqrt{3}/2$, i.e., the same value as for the exponential distribution.

If we consider OPT, looking at the bottom panel of Figure 8, we notice that the discrete distribution maximizing the correlation has, for any k, decreasing probabilities ($p_{1}>p_{2}>\text{⋯}>p_{k}$) and yields a larger correlation than the CIS (for k≥3). We can state that removing the constraint on the support values of the ordinal RV allows it to “adapt” to the skewed continuous distribution better.

We concisely summarize the main results for the Pareto distribution in the following proposition.

Proposition 6 (Pareto distribution.) *For the Pareto distribution with parameter* α>2, *by considering the CIS, the maximum value of the maximal point‐polyserial correlation is obtained through a distribution with decreasing* $p_{i}$ *for* k *smaller than some threshold depending on* α. *By considering OPT, for the same* k≥3, *the maximum value of correlation can be sensibly larger and is obtained through a distribution with decreasing* $p_{i}$. *We observe that, compared to CIS, for the optimal discrete distribution, smaller values are assigned smaller* $p_{i}$ *and larger values are assigned larger* $p_{i}$.

### 4.6 Logistic

The logistic distribution, in its standard version, has PDF $f(x)=\frac{e^{x}}{{\left(1+e^{x}\right)}^{2}}$ and CDF $F(x)=\frac{e^{x}}{1+e^{x}}$, x∈ℝ. The quantile function is $x_{u}=\ln(u/(1-u))$, 0<u<1; moreover, 34𝔼(X)=0 and $\mathrm{Var}(X)=\pi^{2}/3$. Since $\int_{a}^{b}\frac{xe^{x}}{{\left(1+e^{x}\right)}^{2}}dx={\left[\frac{xe^{x}}{1+e^{x}}-\ln(1+e^{x})\right]}_{a}^{b}$, it is easy to derive the expression of the mixed moment between a logistic RV X and a discrete RV $X_{d}$ in the case of comonotonicity and CIS for $X_{d}$:

(17) $${\mathrm{34𝔼}}_{c}(XX_{d})=-\overset{k-1}{\underset{i=1}{\sum }}[F_{i}\cdot \log(F_{i}/(1-F_{i}))+\log(1-F_{i})],$$

and the expression of the correponding maximal point‐polyserial correlation for given probabilities $p_{i}$, i=1,…,k, is then

$$\rho_{PP,\max}=-\overset{k-1}{\underset{i=1}{\sum }}[F_{i}\cdot \log(F_{i}/(1-F_{i}))+\log(1-F_{i})]/\sqrt{\frac{\pi^{2}}{3}\left[\overset{k}{\underset{i=1}{\sum }}i^{2}p_{i}-{\left(\overset{k}{\underset{i=1}{\sum }}ip_{i}\right)}^{2}\right]},$$

which can be maximized with respect to the $p_{i}$ for any k by resorting to the same optimization routines used in the previous subsections.

**FIGURE 9:**
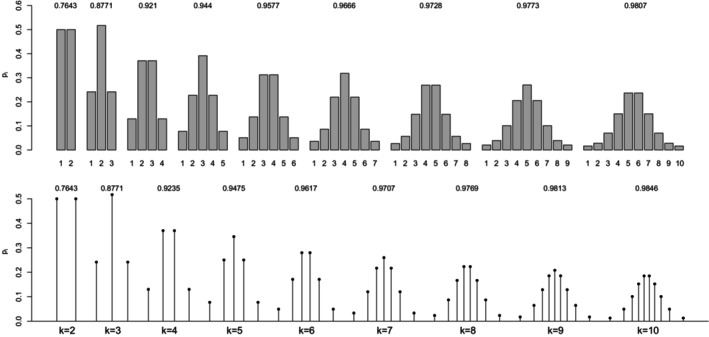
Solution to maximal point‐polyserial correlation problem for logistic distribution for different values of k. In the top panel, we consider the CISs for the ordered categories; in the bottom panel, the support values (OPT) are determined along with the probabilities as a solution to the optimization problem.

Figure 9 displays the k‐point discrete distributions (k=2,…,10) that maximize the maximal point‐polyserial correlation, for CIS (top panel) and OPT (bottom panel). Focusing on the CIS, we note that, as an expected consequence of the symmetry of the logistic distribution, the probability distributions that solve the optimization problem are all symmetrical around the mid‐value (k+1)/2 and unimodal (for k odd) or bimodal (with k even) with the mode(s) coinciding with the central value(s). It is the same situation that occurs with the normal distribution; the only differences are observed in the magnitude of the probabilities $p_{i}$ and of the maximum point‐polyserial correlation. For any value k examined here, the maximum of $\rho_{PP,\max}$ for the logistic distribution is smaller than for the normal distribution.

Let us study the asymptotic behaviour of the maximal point‐polyserial correlation with k in the case of equal‐probability categories; in this case, the expression of the mixed moment (17) specializes into

$$\begin{array}{cc} {𝔼}_{c}{\left(XX_{d}\right)}^{\text{(eq)}} & =\overset{k-1}{\underset{i=1}{\sum }}\left(\ln\frac{k}{k-i}-\frac{i}{k}\ln\frac{i}{k-i}\right)=\overset{k-1}{\underset{i=1}{\sum }}\ln k-\ln(k-i)-\frac{i}{k}\ln i+\frac{i}{k}\ln(k-i)\;\\ & =(k-1)\ln k-\overset{k-1}{\underset{i=1}{\sum }}\frac{i}{k}\ln i+(1-i/k)\ln(k-i);\; \end{array}$$

therefore, the maximal point‐polyserial correlation is given by

$$\rho_{PP,\max}^{\text{(eq)}}=\frac{\left(k-1)\ln k-\overset{k-1}{\underset{i=1}{\sum }}[\frac{i}{k}\ln i+(1-i/k)\ln(k-i)\right]}{\sqrt{\frac{\pi^{2}}{3}\frac{k^{2}-1}{12}}}.$$

Now, for large k, the sum $\sum_{i=1}^{k}\frac{i}{k}\ln i+(1-i/k)\ln(k-i)$ can be approximated by $\frac{1}{k}\int_{0}^{k}x\ln x+(k-x)\ln(k-x)dx={\left[\left(-{\left(k-x\right)}^{2}\ln(k-x)+x(-k+x\ln x))/(2k\right)\right]}_{0}^{k}=k(2\ln k-1)/2$, from which $\rho_{PP,\max}^{\text{(eq)}}$ can be approximated by $\frac{(k-1)\ln k-k\ln k+k/2}{\sqrt{\frac{\pi^{2}(k^{2}-1)}{36}}}$; therefore, its limiting value is $\lim_{k\to \infty }\rho_{PP,\max}^{\text{(eq)}}=3\sqrt{2/\pi^{2}}\approx 0.9549$.

Moving to OPT, inspection of Figure 9 and particularly the bottom panel reveals that the k‐point probability distribution maximizing the correlation is symmetrical around zero for all k. For k≥4, the optimal support values are unequally spaced; the optimal probabilities are different from the homologous probabilities in the CIS case; the resulting maximum correlation is (slightly) larger than for CIS.

We concisely summarize the main results for the logistic distribution in the following proposition.

Proposition 7 (Logistic distribution) *For a logistic distribution, the optimal solution to Problem* (9) *has symmetric probabilities*: $p_{j}=p_{k+1-j},j=1,\text{…},k$. *For the optimal solution to Problem* (10), *for the same* k≥4, *the maximum value of correlation is slightly larger and is obtained through a symmetric distribution with different values for the* $p_{i}$ *and unequally spaced* $x_{i}$.

### 4.7 Power distribution

The CDF and the PDF of the power distribution with parameter α>0, which is a particular case of the Beta distribution, with the second shape parameter β equal to 1, are $F(x)=x^{\alpha }$ and $f(x)=\alpha x^{\alpha -1}$, 0<x<1. When α=1, it reduces to the uniform distribution (Section 4.3). The quantile of level u is $x_{u}=u^{1/\alpha }$. Recalling the expressions for the expectation and the variance of a Beta RV, we have 34𝔼(X)=α/(α+1) and $\mathrm{Var}(X)=\alpha /{\left(\alpha +1\right)}^{2}/(\alpha +2)$.

It is then easy to derive the expression of the value of the mixed moment between a power RV of parameter α and a k‐point discrete RV with tje CIS in the case of comonotonicity, which is given by

$$\begin{array}{l} {\mathrm{34𝔼}}_{c}(XX_{d})=\overset{k}{\underset{i=1}{\sum }}i\int_{F_{i-1}^{1/\alpha }}^{F_{i}^{1/\alpha }}x\alpha x^{\alpha -1}dx=\alpha \overset{k}{\underset{i=1}{\sum }}i\int_{F_{i-1}^{1/\alpha }}^{F_{i}^{1/\alpha }}x^{\alpha }dx=\frac{\alpha }{\alpha +1}\overset{k}{\underset{i=1}{\sum }}i\left[F_{i}^{(\alpha +1)/\alpha }-F_{i-1}^{(\alpha +1)/\alpha }\right]=\frac{\alpha }{\alpha +1}\left[k-\overset{k-1}{\underset{i=1}{\sum }}F_{i}^{(\alpha +1)/\alpha }\right]. \end{array}$$

**FIGURE 10:**
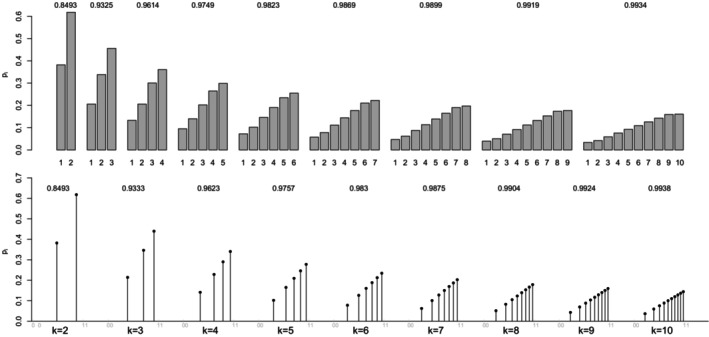
Solution to maximal point‐polyserial correlation problem for a power distribution with parameter α=2 for different values of k. In the top panel, we consider CISs for the ordered categories; in the bottom panel, the support values (OPT) are determined along with the probabilities as a solution to the optimization problem.

The expression of the maximal point‐polyserial correlation can be derived in a straightforward manner. Figure 10 displays the results of its maximization for α=2, with CIS (top panel) and OPT (bottom panel). Focusing on the CIS, we note that for each of the values of k examined and for α=2, the discrete distribution has increasing probabilities, thereby mimicking the increasingness of the PDF of the power RV. $\rho_{PP,\max}$ converges to 1 quite quickly; when k=10, it is equal to .9934, a value just slightly smaller than the corresponding value .9950 obtained for a uniform RV with the same k (Table 2).

Numerical experiments with other values of α>1 show that the optimal solution to (9) does not necessarily respect the condition $p_{i}<p_{i+1}$ for all i≤k−1 and for all k.

Under the equal‐probability setting, the expression of the mixed moment for comonotonic RVs is

$$\begin{array}{l} {\mathrm{34𝔼}}_{c}(XX_{d})=\frac{\alpha }{\alpha +1}\left[k-\overset{k-1}{\underset{i=1}{\sum }}i{\left(\frac{i}{k}\right)}^{(\alpha +1)/\alpha }\right], \end{array}$$

and the maximal point‐polyserial correlation is

(18) $$\max\rho_{PP}^{\text{(eq)}}=\frac{\frac{\alpha }{\alpha +1}\left[k-\frac{k+1}{2}-\sum_{i=1}^{k-1}{\left(\frac{i}{k}\right)}^{(\alpha +1)/\alpha }\right]}{\sqrt{\frac{k^{2}-1}{12}\frac{\alpha }{{\left(\alpha +1\right)}^{2}(\alpha +2)}}}=\frac{\left[k/2-1/2-\sum_{i=1}^{k-1}{\left(\frac{i}{k}\right)}^{(\alpha +1)/\alpha }\right]}{\sqrt{\frac{k^{2}-1}{12\alpha (\alpha +2)}}}.$$

To evaluate the limit of $\rho_{PP,\max}^{\text{(eq)}}$ for k tending to infinity, we can approximate the finite sum in the numerator with $\int_{0}^{1}{\left(\frac{x}{k}\right)}^{(\alpha +1)/\alpha }dx=\alpha k/(2\alpha +1)$. Then the limiting value can be calculated as

(19) $$\underset{k\to \infty }{\lim}\rho_{PP,\max}^{\text{(eq)}}=\sqrt{3\alpha (\alpha +2)}/(2\alpha +1).$$

**FIGURE 11:**
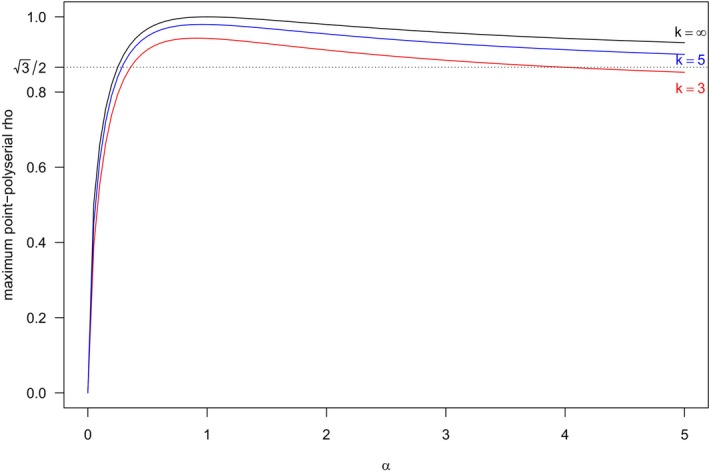
Maximal point‐polyserial correlation between a continuous power RV and a discrete RV with the CIS as a function of the parameter α, for k=3;5;∞, in the case of constant probabilities. The dotted horizontal line indicates the limit, for k and α both tending to ∞, of the maximal point‐polyserial correlation (18).

Note that the limiting value is equal to 1 if and only if α=1, i.e., if we consider a uniform distribution (see also Table 5 for a distribution summary). For all the other positive values of α, (19) is strictly smaller than 1. Figure 11 displays (18) as a function of α for k=3;5;∞. As expected, for a fixed α, the maximal point‐polyserial correlation increases with k. For a given k, the maximal point‐polyserial correlation, now regarded as a function of α, is attained at α=1 (when the power distributions boils down to a standard uniform distribution).

**TABLE 5:** Limits as k tends to +∞ of maximal point‐polyserial correlation in case of equal‐probability categories for k‐point ordinal RV with CIS.

Distribution	$\lim_{k\to \infty }\rho_{PP,\max}^{\text{(eq)}}(k)$
Uniform	1
Normal	$\sqrt{3/\pi }$
Exponential	$\sqrt{3}/2$
Pareto	$\sqrt{3\alpha (\alpha -2)}/(2\alpha -1)$
Logistic	$3\sqrt{2/\pi^{2}}$
Power	$\sqrt{3\alpha (\alpha +2)}/(2\alpha +1)$

Moving to OPT, Figure 10 makes evident that the optimal distribution has still increasing probabilities for any k. Compared to the CIS, the (optimal) support values tend to cluster around the upper bound 1 when k is increasing, with decreasing values of the spacings between consecutive support points. In a symmetrical manner, if we considered a value of α smaller than 1, then we would notice that the (optimal) support values tend to cluster around the lower bound 0 when k is increasing, with increasing values of the spacings between consecutive support points. The gain in correlation with the continuous distribution, with respect to CIS, is, however, negligible. The asymmetry of the distribution, mitigated by the bounded support, does not preclude obtaining high correlation values.

We concisely summarize the main results for the power distribution in the following proposition.

Proposition 8 (Power distribution.) *For a power distribution with parameter* α>0, *the optimal solution to Problem* (9) *has generally increasing (decreasing) probabilities* $p_{i}$ *if* α>1 (α<1), *at least for not too high values of* k *and not too extreme values of* α. *The optimal solution to Problem* (10) *has increasing (decreasing) probabilities* $p_{i}$ *if* α>1 (α<1) *for any* k; *its support values show decreasing (increasing) spacings if* α>1 (α<1). *The improvement in the value of maximum correlation is, however, negligible moving from CIS to OPT*.

## 5 EXAMPLE WITH REAL DATA

Quinn (2004) considered measuring the (latent) political‐economic risk of 62 countries for the year 1987. The political‐economic risk is defined as a country's risk in manipulating economic rules for its own and its constituents' advantage. Quinn (2004) used five mixed‐type variables, namely, the black‐market premium in each country (continuous, used as a proxy for illegal economic activity), productivity as measured by the natural logarithm of the real gross domestic product per worker at 1985 international prices (gdpw2, continuous), the independence of the national judiciary (dichotomous; 1 if the judiciary is judged to be independent and 0 otherwise), and two ordinal variables (both with levels 0<1<2<3<4<5) measuring the lack of expropriation risk (prsexp) and lack of corruption (prscorr). The data set and a complete description thereof can be found in Quinn (2004) or in the R package MCMCpack (Martin et al., 2011). Kadhem and Nikoloulopoulos (2021) applied on this data set a factor model with bivariate copulas that link the latent variable (which can be interpreted as ‘political‐economic certainty’) to each of the observed variables.

Here, we just want to apply the results on maximal point‐polyserial correlation to (a sample drawn from) a bivariate continuous‐ordinal RV; we will consider gdpw2 as the continuous component and prsexp and prscorr as two possible ordinal components, which can be assumed to be the result of ordinalization/discretization of some latent continuous variable. Computations show that the point‐polyserial correlation between gdpw2 and prsexp is .4804; the point‐polyserial correlation between gdpw2 and prscorr is .7250.

Plotting and considering the histogram and boxplot of the empirical distribution of gdpw2 and examining its summary statistics reveals that it is slightly left‐skewed (sample skewness is about −0.433) and platykurtic (sample kurtosis is about 2.10). One can consider fitting a normal and a uniform distribution to these data. Implementing the Kolmogorov–Smirnov test for assessing normality/uniformity for a continuous variable, by adopting the Lilliefors correction to take into account the fact that the parameters must be estimated (Lilliefors, 1967; Novack‐Gottshall & Wang, 2019), we obtain a p‐value equal to .2066 and .034 respectively, which means that the distribution of the continuous variable can be hardly assumed to be uniform but can be more plausibly assumed to be normal.

**FIGURE 12:**
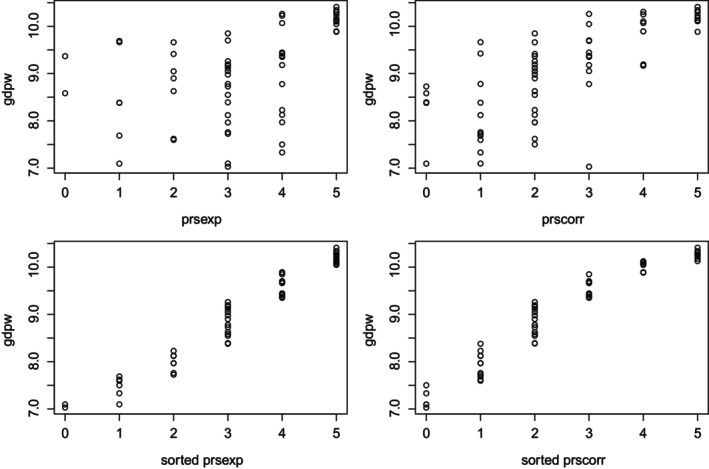
Analysis of real data: scatter plots between continuous and the two ordinal variables before (top panel) and after (bottom panel) reordering. In the latter case, the two pairs of variables are made comonotonic.

Taking the two continuous and marginal distributions as assigned, one can compute the maximal (sample) point‐polyserial correlation by simply computing the correlation between the two samples sorted in ascending order (Demirtas & Hedeker, 2011) (so that the two variables are made comonotonic); see also Figure 12; we obtain .9704 and .9531. These values are quite close to the maximum value obtained between a normal RV and a discrete RV with six categories, which is .9692 (Figure 3); they are slightly smaller than the maximum point‐polyserial correlation between a uniform RV and a discrete RV with six categories, which is .9860 (Table 2).

## 6 MAXIMUM POINT‐POLYSERIAL CORRELATION AS A BASIS FOR DEFINING A *k*‐POINT DISCRETE APPROXIMATION OF A CONTINUOUS RANDOM DISTRIBUTION

The oldest and most popular criterion for constructing a k‐point discrete approximation of an absolutely continuous RV X, with PDF f(x), CDF F(x), expectation μ, and variance $\sigma^{2}$, is based on moment‐matching, i.e., matching as many moments as possible of the continuous RV (provided they exist and are finite). Through a discrete RV sitting on k points, it is possible to match the first 2k−1 positive integer moments; the algorithm that can be used for determining the discrete distribution satisfying this matching is described, for example, in Golub and Welsch (1969); a software implementation, easily adaptable to any continuous distribution, is provided in Toda (2021).

Another way of constructing a k‐point discrete approximation is optimal quantization (Lloyd, 1982), which is based on the minimization of the expected squared distance between X and the closest of the k points. Given k values $x_{1}<x_{2}<\text{⋯}<x_{k}$, we define the expected squared distance or mean squared error (MSE) as

$$\text{MSE}(x_{1},x_{2},\text{…},x_{k})=\mathrm{34𝔼}\min_{x_{1},\text{…},x_{k}\in \mathbb{R}}{\left(x-x_{i}\right)}^{2}=\int_{\mathbb{R}}\min_{x_{1},\text{…},x_{k}\in \mathbb{R}}{\left(x-x_{i}\right)}^{2}f(x)dx.$$

The optimal quantizers ${\tilde{x}}_{1}<\text{⋯}<{\tilde{x}}_{k}$ are the values minimizing $\mathrm{MSE}(x_{1},\text{…},x_{k})$ and can be obtained by rewriting the MSE after introducing k+1 thresholds or cut‐points $c_{i}$, i=0,1,…,k:

$$\text{MSE}(x_{1},x_{2},\text{…},x_{k})=\overset{k}{\underset{i=1}{\sum }}\int_{c_{i-1}}^{c_{i}}{\left(x-x_{i}\right)}^{2}f(x)dx,$$

where the cut point $c_{i}$ is the midpoint between $x_{i}$ and $x_{i+1}$, $c_{i}=(x_{i}+x_{i+1})/2$ for i=1,…,k−1, and $c_{0}=-\infty$, $c_{k}=+\infty$. The k optimal quantizers are also known as *principal points* (Flury, 1990). To each optimal ${\tilde{x}}_{i}$ the probability ${\tilde{p}}_{i}=\int_{c_{i-1}}^{c_{i}}f(x)dx$ remains naturally associated. In Section 4, we proved that the problem of finding the optimal quantizer was fundamentally equivalent to the maximum correlation Problem (10). For more details, one can refer to the recent work by Chakraborty et al. (2021), where the k principal points (k=2,…,8) of several families of random distributions were computed with high numerical precision.

Barbiero and Hitaj (2023) proposed constructing the optimal k‐point approximation to a continuous random distribution as the discrete distribution sitting on k distinct values that minimizes a discrepancy measure (the Cramér, Cramér–von Mises, or Anderson–Darling distance) between the two CDFs; their work is based on that of Kennan (2006), where the author distinguishes the case where the approximating points are assigned a priori, and one needs to compute only the optimal probabilities, from the case were the approximating values are not assigned a priori but must be determined jointly with their probabilities. Barbiero and Hitaj (2021) proposed a similar criterion for constructing a discrete analogue, which is supported over a lattice: ℤ if the continuous RV is real or ℕ if it is positive.

Another alternative to constructing a k‐point approximation to a continuous RV X consists of considering the discrete distribution sitting on the first k natural values that maximizes the maximal point‐polyserial correlation with X, Problem (9), which we discussed in this work.

**FIGURE 13:**
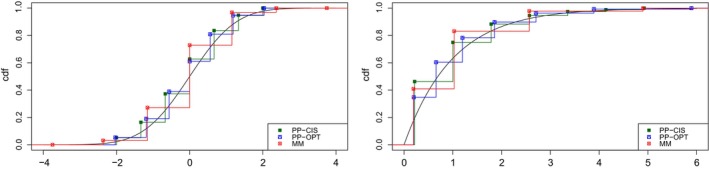
Graphs of CDF of standard normal RV (left panel) and of exponential RV with unit parameter (right panel), along with step‐wise CDFs of their seven‐point approximations derived by maximizing the point‐polyserial correlation in case of CIS (PP‐CIS) and OPT (PP‐OPT) and by moment matching (MM). The last three points of the MM approximation for the exponential RV do not appear in the right panel because they fall outside the x‐axis.

However, rather than considering CISs as the support values, one can adopt an appropriate positive linear transformation thereof, which thus preserves the correlation value. It is reasonable to apply a linear transformation that matches the first two moments of the underlying continuous RV X. Taking this into account, in Table 6, just as a first comparison, for a standard normal RV, we report the k=7 optimal values and corresponding probabilities of the k‐point ordinal RV maximizing the point‐polyserial correlation with X, of the RV obtained as the optimal quantizer of X (the optimal values are directly taken from Chakraborty et al., 2021, table 1, A.9), and of the discrete RV obtained by moment matching (Golub & Welsch, 1969), which preserves the 2·k−1=13 moments of the parent distribution. Analogously, for an exponential RV with unit rate parameter, we report the seven optimal values and probabilities calculated according to the three different approaches (again, for quantization, the optimal values are taken directly from Chakraborty et al., 2021, table 2, A.9). For both continuous distributions, differences across values and probabilities can be easily appreciated and after all were expected, since the criteria behind the different approximations (in particular, if we compare the latter to the former two) are significantly different. Moment matching produces discrete RVs with a larger range and tends to assign very small probabilities to extreme values: One can just look upon the values in the last column of Table 6. To effectively compare the three approximations, Figure 13 displays the CDF of the continuous standard normal (left panel) and exponential RV (right panel) along with the step‐wise CDFs of the discrete RVs.

**TABLE 6:** Optimal 7‐point discrete approximations of a standard normal RV and of an exponential RV with unit parameter.

Standard normal						Exponential with unit parameter					
Max. corr. (CIS)		Max. corr. (OPT)		Moment matching		Max. corr. (CIS)		Max. corr. (OPT)		Moment matching	
Values	Probabilities	Values	Probabilities	Values	Probabilities	Values	Probabilities	Values	Probabilities	Values	Probabilities
−2.000	.0519	−2.033	.0536	−3.750	.0005	.217	.4625	.199	.3479	.193	.4093
−1.333	.1126	−1.188	.1373	−2.367	.0308	1.002	.2865	.657	.2563	1.027	.4218
−.667	.2080	−.561	.1987	−1.154	.2401	1.786	.1338	1.197	.1787	2.568	.1471
0	0	0	.2207	.000	.4571	2.571	.0625	1.857	.1150	4.900	.0206
.667	.2080	.561	.1987	1.154	.2401	3.355	.0292	2.705	.0652	8.182	.0011
1.333	.1126	1.188	.1373	2.367	.0308	4.139	.0136	3.893	.0294	12.734	≈0
2.000	.0519	2.033	.0536	3.750	.0005	4.924	.0119	5.893	.0075	19.396	≈0

## 7 CONCLUSION

The objective of this work was to study the range of the point‐polyserial correlation for several (non‐normal) bivariate distributions and, in particular, determine the maximum attainable value as a function of the distribution parameters of the continuous RV and of the number k of ordered categories of the discrete RV. Finding the expression of the maximal point‐polyserial correlation is often possible since its derivation is related to the availability of closed‐form expressions for partial moments of the continuous distribution. Just as easily, one can find the maximum value of the maximal point‐polyserial correlation, for a given k, numerically (but potentially with precision as high as desired), by using standard constrained optimization routines available in most mathematical and statistical software packages, such as R. Several examples concerning well‐known parametric continuous distributions are detailed and indicate that the maximum point‐polyserial correlation, computed over all k‐point discrete distributions sitting on {1,2,…,k}, is attained at a distribution whose probability values are strictly connected to the continuous random distribution examined: If the continuous distribution is unimodal and symmetrical (e.g., normal and logistic distribution), then the corresponding discrete distribution is unimodal and symmetrical, too; if the continuous distribution is uniform, then the corresponding discrete distribution is a discrete uniform distribution; in the case of a monotone decreasing/increasing PDF (exponential, Pareto, …), then the k probabilities (under some circumstances) are monotone decreasing/increasing as well. From the numerical experiments, it turns out that whatever the continuous distribution is, the maximum point‐polyserial correlation always tends to 1 as the number of categories tends to infinity. We also focused on the equal‐probability setting and determined the limiting value of the maximal point‐polyserial correlation as the number of categories tends to infinity: We find that in all cases, except – as expected – for the uniform distribution, this limiting value is strictly smaller than 1.

In our main analysis, we first assumed that the k ordered categories of the ordinalized RV were assigned the first k CISs. This seemed to be a natural choice, as ordinal variables are standardly handled in this way when it comes to implementing any statistical analysis. However, this can be questioned, and one could consider allowing the scores of the k categories to be unknown and to treat them as additional variables to be optimized (OPT) in order to determine the maximum value of the maximal point‐polyserial correlation. We discovered that when this is done, the problem becomes equivalent to finding the optimal k quantizer or the k principal points of the assigned continuous RV. Using OPT instead of CISs can substantially increase the maximum value of the maximal point‐polyserial correlation, especially if the continuous probability distribution is highly skewed. Moreover, the optimal solution in the case of OPT more closely resembles the behaviour of the continuous distribution in terms of increasing or decreasing trends of the PDF/probabilities.

We emphasize that since the scope of this work was to determine the maximum attainable point‐polyserial correlation between a continuous and an ordinal/discrete RV, our results do not require any assumption about the bivariate continuous RV hypothetically underlying them. If instead one is interested in investigating the attenuation ratio between polyserial and point‐polyserial correlations, as pursued in Bedrick (1995) and Demirtas and Vardar‐Acar (2017), then one must fully specify the bivariate joint distribution or presume some relationship between the two correlations, whose subsistence needs, however, to be carefully checked.

With this in mind, future research will investigate the properties of the k‐point discrete distribution, supported on consecutive integer scores, that maximizes the (maximal) point‐polyserial correlation with an assigned continuous distribution: Are there any cases (in addition to the uniform distribution) for which the probabilities of this discrete distribution can be determined analytically and not just numerically? Can these probabilities be determined analytically as k→∞? Can this discrete distribution be regarded as a valid k‐point approximation of the parent continuous distribution? What are the main differences between this method and other k‐point approximations available in the literature, particularly with optimal quantization, which can be regarded as a generalization thereof?

Another future direction stemmig from this contribution will consider the determination of the minimum attainable point‐polyserial correlation, following the same lines of investigation as in Sections 3 and 4. Such complementary work could be helpful for random generation routines involving mixed‐type data by providing a lower and an upper bound to the correlation between ordinal and continuous variables, which can be required when constructing a huge array of artificial scenarios for the assessment of some mixed‐type data analysis technique, where the level of dependence between two random variables (typically expressed through the correlation coefficient, which remains the most used dependence measure even for mixed‐type data, which recur in psychological, educational, and other behavioural sciences studies) is assigned different values. Being aware of the bounds of the point‐polyserial correlation is also obviously useful if one wants to correctly interpret its sample value on a real data set: Caution is required when interpreting it when the ordinal variables consist of a few categories.

In a nutshell, the utility of this work is twofold. First, it supports the assessment of the maximum attainable correlation between a continuous and an ordinal RV within a modelling/simulation context; second, it provides a possible discrete approximation of a continuous RV, to be used in any application where it is expedient to deal with discrete rather than continuous random distributions.

## AUTHOR CONTRIBUTIONS

**Alessandro Barbiero:** conceptualization; methodology; software; writing – review and editing; writing – original draft; investigation.

## CONFLICT OF INTEREST STATEMENT

The author declares no potential conflicts of interest.

## Supporting information


Appendix: S1

## Data Availability

Data sharing is not applicable to this article as no new data were created or analyzed in this study. R code used for the analyses presented in the paper is available as Supporting Information.
